# Traditional Chinese Medicine for Viral Pneumonia Therapy: Pharmacological Basis and Mechanistic Insights

**DOI:** 10.7150/ijbs.105086

**Published:** 2025-01-06

**Authors:** Yinglu Bai, Tengwen Liu, Shuwen Zhang, Yifan Shi, Yumei Yang, Maoyu Ding, Xiaowei Yang, Shanshan Guo, Xiaolong Xu, Qingquan Liu

**Affiliations:** 1 Beijing Hospital of Traditional Chinese Medicine, Capital Medical University, Beijing 100010, China.; 2 Beijing Institute of Chinese Medicine, Beijing 100010, China.; 3 Chengdu University of Traditional Chinese Medicine, Chengdu, 611137, China.; 4 Clinical Medical College, Beijing University of Chinese Medicine, Beijing 100029, China.; 5 Institute of Chinese Materia Medica, China Academy of Chinese Medical Sciences, Beijing, 100700, China.

**Keywords:** viral pneumonia, traditional Chinese medicine, influenza virus, severe acute respiratory syndrome-coronavirus 2, respiratory syncytial virus, adenovirus

## Abstract

Different respiratory viruses might cause similar symptoms, ranging from mild upper respiratory tract involvement to severe respiratory distress, which can rapidly progress to septic shock, coagulation disorders, and multiorgan failure, ultimately leading to death. The COVID-19 pandemic has shown that predicting clinical outcomes can be challenging because of the complex interactions between the virus and the host. Traditional Chinese medicine (TCM) has distinct benefits in the treatment of respiratory viral illnesses due to its adherence to the principles of “different treatments for the same disease” and “same treatment for different diseases”. This paper examines the effectiveness and underlying mechanisms of key TCM treatments for viral pneumonia in recent years. The aim of this study was to discover and confirm the active substances of TCM with potential therapeutic effects on viral pneumonia and their integrative effects and synergistic mechanisms and to provide a scientific basis for elucidating the effectiveness of TCM treatment and drug discovery. Furthermore, a thorough analysis of previous research is necessary to evaluate the effectiveness of TCM in treating viral pneumonia.

## Introduction

Viral pneumonia is a condition in which a viral infection spreads from the upper respiratory tract to the lungs, causing inflammation and impairing lung function. Viral pneumonia is mainly caused by viral infections in the upper respiratory tract, the reactivation of dormant viruses, and the presence of viruses in the bloodstream. Recently, viral pneumonia caused by pathogens such as severe acute respiratory syndrome-coronavirus 2 (SARS-CoV-2), influenza virus (IFV), respiratory syncytial virus (RSV) and adenovirus (AdV) has become a pressing public health issue worldwide.

Antiviral treatment is essential, but human respiratory viruses are in high diversity and it poses challenges for drug development. Currently, treatment for the majority of respiratory viral infections is not specific, and there are limited options for clinically specific antiviral therapy, with drugs such as abidol, oseltamivir, zanamivir, and peramivir being the main options for IFV. At present, there are limited medications authorized for treating SARS-CoV-2^1^. Although RSV and AdV are associated with a high disease burden, there is a lack of specific and nonspecific methods for treating or preventing infection. New medications must continue to be created to combat emerging strains of respiratory viruses in anticipation of future pandemics, with the goal of decreasing dosing frequency and minimizing adverse reactions from drug interactions.

The extensive diversity of viruses and intricate pathogenic processes have long been primary areas of study in the life sciences and medical fields. With the global increase in viral respiratory diseases, traditional Chinese medicine (TCM) has shown unique advantages and benefits in the treatment of various pandemic viral pneumonias. An increasing number of high-quality clinical studies indicate that TCM has therapeutic effects in treating viral pneumonia(Oseltamivir compared with the Chinese traditional therapy maxingshigan-yinqiaosan in the treatment of H1N1 influenza: a randomized trial/ Effectiveness and safety of Sanhan Huashigranules versus nirmatrelvir-ritonavir inadult patients with COVID-19: Arandomized, open-label, multicenter trial/ Combination of Hua Shi Bai Du granule (Q-14) and standard care in thetreatment of patients with coronavirus disease 2019 (COVID-19): A singlecenter, open-label, randomized controlled trial). This paper will examine how TCM has been used to treat various viral pneumonia outbreaks in recent years, outlining the mechanisms and pharmacological foundations of TCM in preventing and treating viral pneumonia. The goal is to provide a scientific foundation for the clinical use of TCM.

## 1. Molecular mechanism of viral pneumonia and treatment

Viruses must live inside host cells and rely on the metabolic processes of host cells to reproduce. Viruses are composed of genetic material enclosed by a protein shell, known as a capsid, which may be enveloped in a lipid layer. Viruses are categorized based on their nucleic acid genome, capsid shape, presence of a lipid envelope, replication method, tropism, and the pathology they induce. This review provides an overview of possible medications and traditional Chinese remedies that can be used treat viral pneumonia by targeting various viruses (Table S1).

### 1.1 IFV

Influenza viruses are part of the *Orthomyxoviridae* family and are enveloped viruses with a negative RNA genome. Its genome is segmented into eight fragments, which provides it with evolutionary advantages (A functional sequence-specific interaction between influenza A virus genomic RNA segments). These viruses are divided into four types, A-D, among which the A, B, and C influenza viruses can infect humans and cause disease. The viral proteins polymerase basic protein 1, polymerase basic protein 2, and polymerase acidic protein combine to create an RNA-dependent RNA polymerase (RdRp) complex responsible for transcribing and replicating the viral genetic material. The binding of nucleoprotein (NP) to viral RNA is essential for transcription and replication processes. The virion surface contains three essential membrane proteins: haemagglutinin (HA), neuraminidase (NA), and matrix 2 ion channel (M2).

### 1.2 SARS-CoV-2

There are three methods through which coronaviruses can enter host cells: receptor-mediated plasma membrane fusion, receptor-mediated endocytosis, and antibody-dependent viral entry. The single-stranded RNA-enveloped virus SARS-CoV-2 infects cells by using its structural spike (S) protein to attach to the angiotensin-converting enzyme 2 (ACE2) receptor. After binding, the virus particle enters cells by utilizing host cell receptors and endosomes. The S protein can enter cells with the help of a type 2 transmembrane serine protease known as TMPRSS2. After entering the cell, viral polyproteins that contain the replicase-transcriptase complex are produced. Subsequently, the virus uses its RdRp to produce RNA. The production of structural proteins results in the finalization of viral particles and their subsequent release^2^-^4^. Potential targets for drug therapy have been identified for various stages of the viral lifecycle. Previous research has identified the S protein, ACE2, TMPRSS2, 3C-like protease (3CLpro), RdRp, and papain-like protease (PLpro) as key targets for antiviral medications in the treatment of coronavirus infections^5^, ^6^.

### 1.3 RSV

RSV is a single-stranded RNA virus belonging to the *Paramyxoviridae* family and Pneumovirus genus. RSV infection is mainly mediated by glycoproteins F and G. Adhesion proteins (G) adhere to host cell membranes, promoting viral adsorption on the cell surface, and fusion proteins (F) mediate fusion of the viral envelope with host cell membranes, allowing the virus to enter the cell. During viral fusion and entry, the F protein changes from a substable prefusion conformation to a stable postfusion conformation. The F and G proteins are highly immunogenic and stimulate the production of serum-neutralizing antibodies. F proteins have specific antigenic determinants outside the viral envelope, and they can cause significant conformational changes in membrane fusion due to their conserved intersubtype sequences and their key role in the membrane fusion process.

### 1.4 AdV

Adenoviruses lack an envelope and contain a linear double-stranded DNA genome ranging from 26 to 45 kilobase pairs. Virus particles consist of a core portion of DNA and a protein coat surrounding it^7^. The protein coat (icosahedral shell) consists mainly of 240 hexa-coordinated capsomeres (12 on each triangular face of the icosahedron), 12 penta-coordinated capsomeres, and 12 fibrillar proteins extending vertically from the penta-coordinated capsomeres. Each hexa-coordinated capsomere is a homotrimer of proteins, and the hexa-coordinated capsomeres consists of a pentahedral base and a triangular tip. The basal portion within the capsid has antigenic sites common to all HAdV phenotypes, which are not able to induce neutralizing antibodies because they are all located internally.

## 2. Characteristics of TCM syndrome elements of viral pneumonia

The fundamental concept of TCM is centred on treating the body as a whole and using syndrome differentiation to diagnose and treat illnesses, including viral infections. Holism views the body as a unified entity, linking the onset and progression of specific ailments to overall health. Therefore, addressing the entire body is essential for the effective treatment of localized diseases. Syndrome differentiation and treatment are described in the following manner. Syndrome differentiation involves identifying and categorizing the specific illness, including understanding its location, cause, characteristics, and balance between opposing forces within the body, all of which reveal underlying changes in health.

Data were collected from various sources, including PubMed, the Web of Knowledge, CNKI, the Wanfang database, VIP database and CBMdisc, between January 1, 2000, and April 29, 2024, to gather research on the prevalence of evidence for the use of Chinese medicine to treat IAV, IBV, AdV, RSV, and SARS-CoV-2 infections. A search was conducted for studies related to the TCM syndrome types of viral pneumonia. There were a total of 13 studies related to IVA, 14 related to IVB, 18 related to RSV, 20 related to AdV, and 55 related to SARS-CoV-2. The cases were mainly from 23 provinces, municipalities and autonomous regions in China, including Wuhan, Beijing and Guangzhou, and Saudi Arabia.

**Figure 1:**
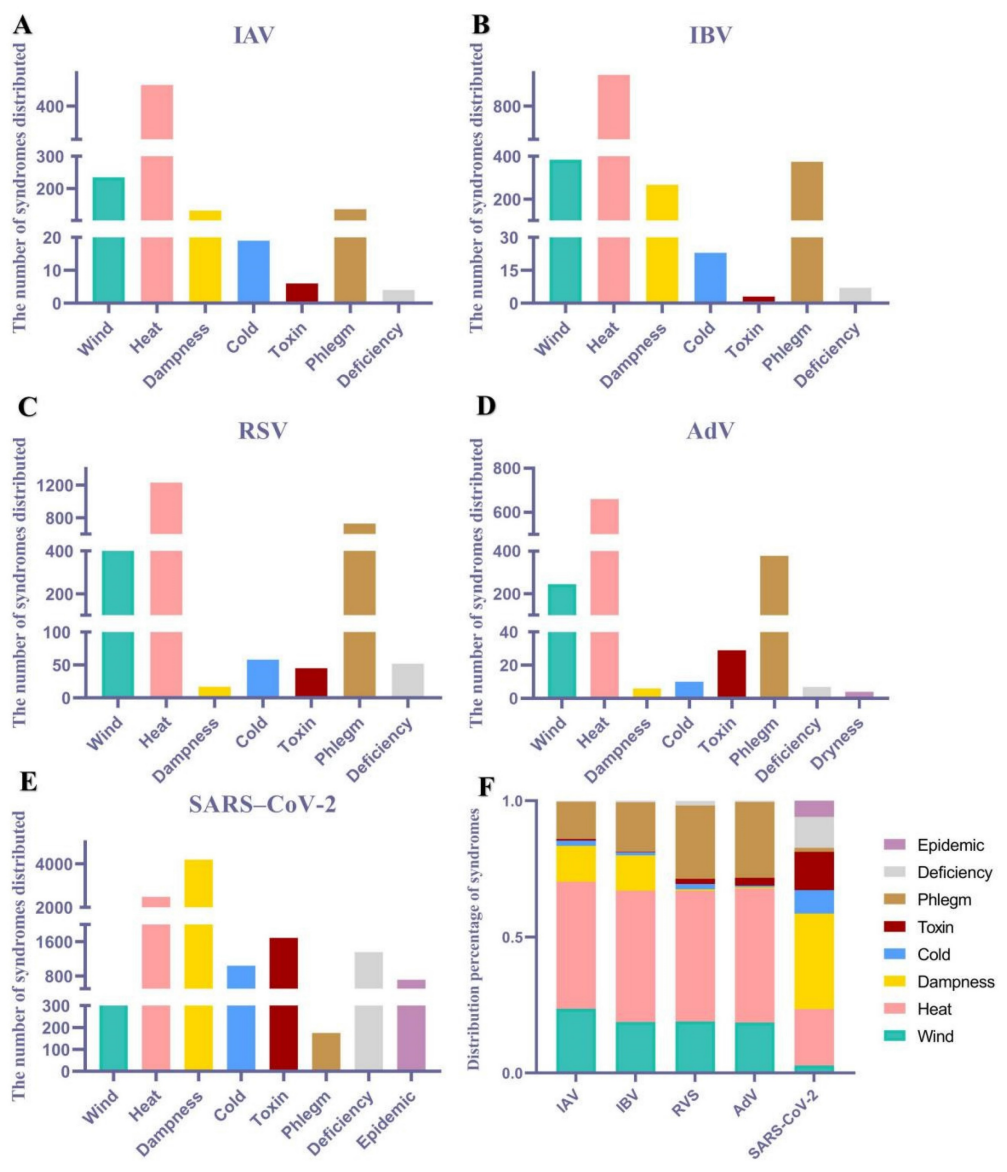
Distribution of syndrome elements associated with viral pneumonia. The syndromes distributed of (A) IAV, (B) IBV, (C) RSV, (D) AdV, (E) SARS-CoV-2. (F) Distribution percentage of TCM syndrome of multiple viral pneumonia.

Content related to TCM syndromes from the included literature was extracted, and an Excel spreadsheet was created for data organization. Syndrome elements were extracted and dissected, for example, "Wind-Heat Assailing the Defensive Level" and "Wind-Heat Blocking the Lungs," were uniformly extracted as "Wind-Heat" and then dissected into two syndrome elements: "Wind" and "Heat." According to the statistical results, a total of eight pathological syndrome elements, namely, wind, heat, dampness, cold, toxin, phlegm, dryness, and epidemic, were dissected. The percentage was calculated as follows: frequency of pathological syndrome elements (%) = number of occurrences of the pathological syndrome element/total number of occurrences of all pathological syndrome elements × 100%. Based on the statistical results, the top two elements for both IVA and IVB were wind and heat; for RSV and AdV, the top two elements were heat and phlegm; and for SARS-CoV-2, the top two elements were heat and dampness (Fig. 1).

In this paper, we chose 13 representative TCM formulas for viral pneumonia (e.g., Dayuanyin, Qingfei Paidu decoction and Jinhua Qinggan granules) and analysed the efficacy of their constituent TCM medicines based on the statistical results of a total of eight pathological syndromes, namely, wind, heat, dampness, cold, toxin, phlegm, dryness, and epidemiology (Table 1). IFV, SARS-CoV-2, RSV and AdV had the most drugs for treating heat syndrome, followed by drugs for treating the symptomatic syndromes of phlegm, dampness, wind and toxin, and the pattern of their use was consistent with the trend of TCM evidence for viral pneumonia, suggesting that TCM practitioners can adopt the same or different treatments according to similarities and differences in the constituent elements of diseases. In addition, this paper provides an overview of the history, efficacy and treatment and clinical studies of various Chinese herbal formulas (Table 2).

## 3. Pharmacological substance and mechanisms of TCMs against respiratory viruses

In this paper, we selected clinically representative Chinese medicines and their active ingredients and systematically reviewed their mechanisms of action and the material basis of their efficacy in the treatment of viral pneumonia (Tables 3, 4). The results showed that clinically effective formulas treat viral pneumonia by interfering with respiratory viral infection, regulating host immune function, protecting organs, regulating metabolism, and regulating multi-pathway interactions.

### 3.1 Antiviral intervention using TCMs

Viruses can directly damage host cells by entering them and replicating at the expense of the host. Viruses possess specific cell surface proteins that bind to particular host cell surface proteins. Viral replication within cells is dependent on cell type-specific transcription factors that identify viral enhancers and promoter elements. After entering host cells, viruses can harm or destroy cells through various methods. AdVs cause direct destruction of infected cells, whereas IAVs and CoVs trigger apoptosis in their target cells. AdV and RSV infections lead to the development of inclusion bodies, while syncytium formation has been observed in cells infected with RSVs and CoVs. TCMs directly inhibit respiratory viruses by disrupting viral entry, replication, assembly, and release.

#### 3.1.1 Inhibition of viral invasion by TCMs

After IFV infection, patchouli alcohol significantly inhibited the replication of various IAV strains in a laboratory setting, potentially preventing virus infection by deactivating virus particles and disrupting the early stages after virus attachment, ultimately hindering the expression of the viral proteins HA and NP^48^, ^49^.

During the invasion of SARS-CoV-2, Danshensu exhibited strong antiviral effects against the virus, with an EC50 value of 0.97 μM; it also successfully blocked the entry of the SARS-CoV-2 S protein pseudotyped virus into ACE2-overexpressing HEK-293T cells (IC50 = 0.31 μM) and Vero-E6 cells (IC50 = 4.97 μM), as reported by W. Wang *et al.* in 2022^50^. Kaempferol suppressed the invasion of SARS-CoV-2 in laboratory and animal studies by inhibiting viral fusion and interacting with HR regions of SARS-CoV-2 S2 subunits, as reported by Gao *et al.* in 2023^51^. Ephedrine has the ability to block the entry of SARS-CoV-2 S into ACE2h cells by decreasing the entry rate of pseudoviruses in a pseudovirus model^52^. Li J and colleagues demonstrated that preincubation of Lenti-S virus, rather than the host cells, with glycyrrhizic acid resulted in decreased Lenti-S infection, suggesting that glycyrrhizic acid specifically targeted the virus. Surface plasmon resonance analysis indicated that GA hindered the interaction between a recombinant S protein and host cells, as demonstrated by J. Li and colleagues in 2021^53^. Mangiferin treatment significantly downregulated the expression of the ACE2 and TMPRRS2 genes. According to Spampinato and colleagues, mangiferin effectively inhibited virus entry^54^. In their research, Wan, Lina, and colleagues investigated SARS-CoV-2 pseudoviruses. They discovered that the combined use of andrographolide and baicalein, compared with using each compound alone, had a notable impact on reducing the angiotensin-converting enzyme 2 protein level and the entry of SARS-CoV-2 into cells. Additionally, this combination inhibited the primary protease activity of SARS-CoV-2^55^.

During RSV infection, 18β-glycyrrhetinic acid exhibited strong anti-HRSV effects. Pretreatment with Glycyrrhiza glabra yielded greater efficacy (*p*<0.0001) in preventing viral attachment (*p*<0.0001) and entry (*p*<0.0001) into host cells, as suggested by Feng Yeh *et al.*, 2013^56^.

#### 3.1.2 Inhibition of viral replication by TCMs

In an *in vitro* study, Utsunomiya *et al.* discovered that the inclusion of caffeic acid within 3 h of infection yielded strong antiviral effects, indicating that the compound targets early infection stages^57^. Sodium baicalin had a pronounced inhibitory effect on NAs. Jin *et al.* reported that the concentration of sodium baicalin needed to inhibit 50% of the activity of H1N1-H275Y and cells expressing the NA protein of A/Anhui/1/2013-R294K (H7N9-R294K) was 214.4 μM and 216.3 μM, respectively^58^. In their study, Wang QW and colleagues delved into the impact of rhein, a key component of rhubarb, and discovered that rhein effectively hinders the adsorption and replication of IAVs; however, it does not have a substantial effect on inactivating IAVs or on cells prior to infection^59^.

Zhang, Ya-Ni and colleagues discovered that gallocatechin and sciadopitysin exhibited the strongest inhibitory effects on SARS-CoV-2 3CLpro, with IC50 values of 0.98 μM and 3.21 μM, respectively^60^. According to the results reported by Zandi and colleagues, baicalein and baicalin were found to directly inhibit SARS-CoV-2 RdRp and suppress its activity. Baicalein demonstrated greater potency than did baicalin in this inhibition^61^. During viral load reduction experiments, honokiol was shown to reduce both viral RNA copy numbers and viral infectious progeny titres. This substance also blocked the replication of SARS-CoV-2 in human A549 cells expressing ACE2 and transmembrane protease serine 2. Honokiol was found to inhibit virus replication at a stage following entry into the replication cycle, as demonstrated by time-of-addition and other assays. Honokiol showed effectiveness against newer strains of SARS-CoV-2, such as Omicron, and blocked the activity of various human coronaviruses^62^. Linoleic acid exhibited a strong affinity to SARS-CoV-2 RdRp by directly binding to the cavity created by the RNA double helix and protein^63^.

A 2016 study by H. Shi and colleagues revealed that the addition of 10 and 30 μM baicalein during the RSV viral replication process led to a notable reduction in phagolocalization, indicating that baicalein can hinder RSV replication^64^. *In vitro*, licochalcone A can hinder RSV replication and alleviate cell damage caused by RSV. Additionally, *in vivo* studies have shown that licochalcone A can protect infected mice by decreasing viral levels and inflammation in the lungs^52^. In a study by Siyi Che and colleagues, andrographolide partially blocked RSV replication by increasing HO-1 expression but did not trigger the antiviral interferon response^65^.

#### 3.1.3 Inhibition of virus release by TCMs

In IFV infection, chlorogenic acid targets NA proteins to inhibit the release and spread of progeny virus particles. Chlorogenic acid inhibited H1N1 viral NA more than H3N2 viral NA. In addition, chlorogenic acid inhibits a variety of oseltamivir-resistant strains^66^. Caffeic acid has been shown to effectively block the damage caused by the virus and prevent cell death, indicating its ability to protect virus-infected cells^57^.

Yang, L *et al.* established that in SARS-CoV-2 infection, IgG ICs induce macrophage pyroptosis by upregulating the expression of GSDME and GSDMD through CEBP-δ activation, while verbenalin inhibits this process. Verbenaside has been shown to have a therapeutic impact on lung damage^67^.

In shielding the host from harm to cells, licochalcone A treatment for RSV infection has a dual impact. Licochalcone A can trigger the Nrf2/HO-1 pathway in a manner that does not depend on Keap1, ultimately preventing oxidative stress caused by ROS in models of infection with RSV^52^.

### 3.2 Immune-mediated protection induced by TCMs

In normal lungs, local immune cells like alveolar macrophages, conventional and plasmacytoid dendritic cells, as well as tissue-resident lymphocytes and eosinophils, keep an eye on the tissue for external dangers. Moreover, club cells and goblet cells, which are types of respiratory epithelial cells, produce mucins, surfactants, and other substances to preserve homeostasis and ensure immune cells remain dormant. In viral infections, innate immune receptors, also known as pattern recognition receptors, detect viruses and trigger immune responses. The release of chemokines and growth factors by the respiratory epithelium and immune cells in the local area results in the sequential attraction and stimulation of neutrophils, monocytes, NK cells, and T cells. IFN-α/β and IFN-λ are the main cytokines released in response to viral detection, along with other inflammatory cytokines and ISGs. As viral infection progresses and triggers the immune system, it may result in significant lung injury and additional inflammation throughout the body. Further lung damage can lead to hypoxia, acute respiratory distress syndrome, asthma, structural remodelling of the lungs, and, in extreme cases, even organ failure or death.

TCMs have been used to treat respiratory infectious diseases for thousands of years. Research has shown that TCMs can treat respiratory viral infections by boosting the immune system and restoring balance in the body with the help of anti-inflammatory cytokines, immunosuppressive molecules, and efferocytosis to eliminate harmful immune cells and promote tissue healing.

#### 3.2.1 Modulation of innate immunity by TCMs

Innate immunity plays a pivotal role as the first line of defense against viral infections, and TCMs have demonstrated profound effects in modulating these mechanisms. Through targeting key pattern recognition pathways and reducing excessive inflammatory responses, TCMs help maintain immune balance and prevent tissue damage.

Within the context of IFVs, the administration of Gegen Qinlian decoction (GQD) has been shown to suppress certain crucial components of the TLR signalling pathway, including TLR7, MyD88, and NF-κB p65. Ultimately, GQD triggers a well-regulated inflammatory reaction in the body to reduce immune-related damage and enhance overall clinical and survival outcomes^68^. The Qingwenzhike prescription (QWZK) lowers the white blood cell count and neutrophil count in bronchoalveolar lavage fluid (BALF) while also increasing lymphocyte and monocyte counts; additionally, it can suppress the progression of lipopolysaccharide (LPS)-induced acute lung injury (ALI). The effect of QWZK on ALI may involve the suppression of the TLR4/NF-kB pathway and NLRP3 inflammasome activation, leading to reductions in TLR4, p-IKKα/β, p-IκBα, p-NF-κB, NLRP3, cleaved caspase-1 and ASC expression^33^. They target toll-like receptors (TLRs) involved in recognizing viruses, specifically TLR3 for double-stranded RNA and TLR7/8 for single-stranded RNA, functioning through a TRIF-dependent signaling pathway. Respiratory epithelial cells and immune cells contain these receptors. PRRs are initiated upon viral recognition, leading to the release of type I and type III interferons and cytokines that help prevent and clear respiratory viral infections.

Jinhua Qinggan granules (JHQG) play a role in enhancing neutrophil apoptosis through the intrinsic mitochondrial apoptotic pathway. JHQG treatment has been shown to significantly reduce the levels of TNF-α, IL-1β, and IL-6 in mice induced with LPS^69^. In a study by D. Shi *et al.* in 2023, Qingfei Paidu decoction (QFPDD) was found to suppress inflammatory cytokines in LPS-stimulated macrophages, ameliorate ALI in mice, and increase the survival rate of mice exposed to a lethal dose of LPS^70^. Furthermore, QFPDD suppressed the activation of M1 macrophages and reduced the levels of IL-6, TNF-α, MIP-2, MCP-1, and IP-10 while increasing IL-10 expression, as reported by Ye *et al.* in 2023^71^. When a virus is detected, the lung epithelium releases cytokines such as TNF-a, IL-6, IL-1b, G-CSF, and GM-CSF, which play a crucial role in modulating the immune response, regulating cell proliferation and maturation, and managing viral transmission. Airway epithelial cells contribute to bodily homeostasis by modulating lung inflammation. Cells regulate IL-1b responses in a steady state by secreting IL-1RA and IL-1RII inhibitors. During a viral lung infection, the anti-inflammatory state can quickly shift due to the release of pro-inflammatory cytokines. JHQG significantly reduced lung injury in a mouse model of SARS-CoV-2 infection by inhibiting macrophage activation, decreasing the levels of proinflammatory mediators, suppressing the expression of p-ERK and p-STAT3, and inhibiting TLR4/NF-κB activation. The main active ingredient of JHQG was identified as luteolin through a combination of network pharmacology and HPLC. Luteolin interacts with the TLR4/MD2 complex, leading to anti-inflammatory effects and protection against ALI^72^. Further studies revealed that XFBD inhibits acute inflammatory responses by downregulating the IL-6/STAT3 pathway, controlling macrophage activity, and reducing inflammatory cytokine production. These mechanisms not only suppress acute inflammation but also prevent long-term damage, such as pulmonary fibrosis caused by innate immune overactivation^73^, ^74^. *Paramyxoviruses* effectively counteract innate cellular immunity mechanisms. Numerous encode the V protein, an immune evasion protein that disrupts RNA recognition in the cytoplasm, thereby inhibiting IFN production and the antiviral response. V proteins are recognized for their capacity to disrupt STAT proteins, thereby inhibiting ISG expression. These proteins additionally engage with RIG-I-like receptors.^75^, ^76^. Coronaviruses exhibit differing capacities to suppress ISG expression in reaction to IFN signaling via various nonstructural and accessory proteins. Notably, nsp1 inhibit STAT phosphorylation in a virus-specific manner, with SARS-CoV-2 proteins being the most potent inhibitors^77^, ^78^.

Xuanfei Baidu Decoction (XFBD) controls immune responses mediated by neutrophils, specifically regulating the formation of NETs through the CXCL2/CXCR2 axis. According to Zhou *et al.*, XFBD can alleviate ALI during the clinical course by targeting neutrophils and inhibiting their infiltration^79^. After viral infection, macrophage inflammatory protein 1b or IL-8 recruits neutrophils, leading to lung inflammation. Activated endothelial cells also produce pro-inflammatory cytokines, chemokines, and interferons (e.g. IL-1b, CXCL9, and IFN)^80^, ^81^, as well as adhesion molecules (e.g. ICAM-1 and VCAM) that recruit white blood cells to the site of infection and mediate white blood cell/endothelial cell adhesion.

#### 3.2.2 Modulation of adaptive immunity by TCMs

The adaptive immune system's response to the complete removal of viruses and the formation of memory is accomplished through B cells and T cells.B cells can produce neutralizing virus particles that clear virus-infected cells. Herbal medicine shows the potential to modulate cellular and humoral immune responses. These effects are mediated by regulating T cells, B cells and cytokines to promote immune homeostasis and recovery.

GeGen QinLian decoction (GQD) treatment for IFV infections was shown to yield systemic protection by inhibiting the inflammatory differentiation of CD4+ T cells and influencing the expression of inflammatory cytokines in mesenteric lymph nodes (mLNs) and serum, as reported by Deng and colleagues in 2021^82^. CD4+ follicular helper T cells (TFH) play a crucial role in initiating an effective B-cell response during infections by forming germinal centers in secondary lymphoid tissues, which are essential for B-cell maturation, proliferation, and memory development. Elevated TFH cell counts correlate with increased levels of influenza-specific IgM and IgG antibodies following vaccination^83^ .

QFPDD markedly increases the number of immune cells in the peripheral blood of mice with pneumonia caused by SARS-CoV-2 while also reducing the concentrations of proinflammatory cytokines in the lungs. The genes upregulated by QFPDD are enriched in SRP-dependent cotranslational proteins that focus on membrane targeting, positive regulation of lymphocyte differentiation, lymphocyte activation, B-cell differentiation, and CD4-positive, alpha-beta T cell differentiation^84^. Adoptively transferred CD4+ T cells in mice lacking mature T cells promote an effective antibody response to protect from an IAV infection, but not in SCID mice lacking both T cells and B cells. Depletion of CD4+ T cells also correlated with decreased antibody responses during SARS infection^85^.

The greater proportion of CD4+ and CD8+ T cells in the blood of subjects who received Shufeng Jiedu capsules (SFJDC) suggests that this treatment may help reduce or even prevent lymphopenia induced by SARS-CoV-2. Furthermore, Xia and colleagues reported that SFJDC reduced the levels of the inflammatory markers Il-6, IL-10, TNF-α, and IFN-γ in lung tissue^86^. An analysis of blood samples from critical COVID-19 patients revealed the presence of SARS-CoV-2 spike glycoprotein-specific CD4+ T cells in all patients, while 80% exhibited specific CD8+ T cells, which emerged early in the infection and increased over time^87^.

Baicalin treatment during RSV infection was shown to lead to the decreased infiltration of T lymphocytes and decreased expression of proinflammatory factors, with a moderate reduction in RSV titres in lung tissues^64^.

Overall, these findings illustrate the diverse mechanisms through which TCMs modulate adaptive immunity, emphasizing their potential as complementary therapies in managing viral infections and immune dysregulation.

### 3.3 Regulation of metabolism by TCMs

Viruses are the most prevalent and diverse biological entities globally, possessing a vast range of genetic material and the capability to infect multiple species.^88^. Through coevolution, viruses have evolved diverse mechanisms to enhance their replication.A mechanism involves altering host metabolism by disrupting key metabolic pathways and targeting master regulatory proteins. Metabolic signaling pathways are crucial for coordinating cell signaling and gene transcription, making their precise modulation essential for organisms. Consequently, viruses have adapted to manipulate these pathways and modify metabolism.

Infection disrupts mitochondrial function, resulting in a lack of energy that is then offset by an increase in glycolysis^89^. AMPK, a universally present cellular energy detector in eukaryotic cells, is a crucial target for numerous viruses^90^; it activates hypoxia-responsive factor 1 (HRF-1), leading to an increase in glucose absorption and glycolysis-promoting enzymes. MXSGD treatment successfully decreases the amount of virus inside cells and decreases the levels of ROS, overall iron, and ferrous ions; it also improves mitochondrial function and blocks the activation of cellular ferroptosis and the HIF-1 signalling pathway^91^. Viruses alter glucose metabolism to enhance energy availability and facilitate their replication by adjusting specific signaling pathways. Many viruses achieve this by triggering aerobic glycolysis, referred to as the Warburg effect.^92^. The Warburg effect involves the conversion of pyruvate to lactate via lactate dehydrogenase (LDH) at the end of glycolysis, despite the presence of oxygen. It regulates key enzymes involved in aerobic glycolysis, including glucose transporters, hexokinase, phosphofructokinase, pyruvate kinase, and LDH. Aerobic glycolysis leads to increased lactic acid production, reduced glycolytic intermediates for the TCA cycle, and elevated glucose consumption^93^ .

Glutaminolysis involves the use of glutamine to produce TCA cycle intermediates when pyruvate is unavailable. Anaplerosis through glutamine is needed for viral replication^94^. QFPDD impacts the TCA cycle and fatty acid metabolism pathways in the liver by increasing the levels of malonic acid and adenosine monophosphate. QFPDD has a significant impact on the regulation of purine metabolism in the liver; this finding was validated through an analysis of single-cell RNA sequencing data. QFPDD has been found to regulate the expression of key genes linked to metabolic pathways, potentially leading to improved immune functions^84^. Glutamine serves as an alternative energy source by contributing to the TCA cycle, which generates NADPH for the electron transport chain. The disconnection between glycolysis and the TCA cycle in viral life cycles that induce aerobic glycolysis leads to a reliance on glutamine. Viral replication was significantly reduced in cells cultured without glutamine, highlighting its necessity for optimal viral survival. Moreover, QFPDD's role in restoring gut microbiota diversity and abundance has been highlighted in studies integrating microbiome and metabolome analyses. These studies showed that QFPDD increases the richness of beneficial genera such as Alistipes and Odoribacter while modulating lipid metabolism, including glycerophospholipids and fatty acids, which strongly correlate with immune-inflammatory markers. This evidence supports the existence of a microbiota-metabolism-immune axis central to QFPDD's therapeutic effects^95^. Additionally, QFPDD has been found to ameliorate aberrant cell-cell communication and purine metabolism disorders in the liver, reducing systemic inflammation and liver injury in viral pneumonia models^84^.

DYY greatly enhances the overall condition and lung tissue structure of mice with lung syndrome caused by RSV and cold dampness, resulting in a decreased lung index and reduced levels of IL-6 and IL-1β in the lungs. Thirty-five potential biomarkers have been identified as being associated with the regulatory impact of DYY, primarily linked to purine metabolism, arachidonic acid metabolism, and glycine, serine, and threonine metabolism^96^. Similarly, Gegen Qinlian decoction (GQD) has shown notable efficacy in reversing metabolic dysfunction in lipopolysaccharide-induced acute lung injury (ALI). Pathway analysis has revealed that GQD restores phenylalanine, tyrosine, and tryptophan metabolism as well as glycine and lysine pathways, alleviating amino acid and energy metabolism disorders caused by ALI^97^. Lipid droplets (LDs) are common organelles characterized by a core of neutral lipids encased in a phospholipid monolayer.^98^. TAGs are released through lipolysis when activated, usually during cell growth or nutrient depletion^99^. Diacylglycerol O-acyltransferase 1 and 2 are key regulators of LD formation. These enzymes attach to the ER membrane, overseeing TAG synthesis and packaging within the phospholipid bilayer, leading to LD budding and subsequent release into the cytoplasm.^99^.

In addition, kaempferol, a flavonoid found in various TCM formulations, further exemplifies the metabolic regulatory potential of TCMs. Kaempferol alleviates oxidative stress and inflammation by inhibiting TLR4/MyD88-mediated NF-κB and MAPK signaling pathways. It also improves antioxidant defenses by increasing superoxide dismutase (SOD) activity and reducing ROS and lipid peroxidation. These properties position kaempferol as an effective agent in managing pulmonary metabolic disturbances induced by viral infections, such as H9N2 influenza virus-induced ALI^100^.

### 3.4 Protection of organs by TCMs

The use of the Maxing Shigan Decoction (MXSG) significantly increases body mass, the spleen index, and the thymus index while decreasing the lung index. MXSG also reduces IL-8 and IFN-γ levels as well as the protein and mRNA levels of JAK1, JAK2, STAT1, IRF9, and IFN-γ in lung tissue; decreases the protein and mRNA levels of JAK2, STAT1, and IRF9 in colon tissue; and helps alleviate pathological damage in lung and colon tissues^101^. Yinqiao anti-infective powder (YQAIP) has been shown to enhance lung tissue inflammation and pathology in IVP mice, leading to increased survival, lower lung index and pneumonia factor levels, a lower lung viral load, and the suppression of the key mitochondrial genes *Pnpt1*, *Mthfd2*, and *Lactb*^102^. In mice, Dayuanyin (DYY) led to a notable decrease in right ventricular systolic pressure, alleviated lung damage, and lowered the levels of inflammatory markers; additionally, it blocked the activation of the NF-κB signalling pathway induced by hypoxia^103^.

QFPDD has the potential to ameliorate IFV-induced heart damage by decreasing cell necroptosis and apoptosis, suppressing inflammation, and reducing the levels of HIF-1α^104^.

QFPDD shields the liver from damage by elevating the levels of adenosine and inosine. QFPDD therapy markedly increases the proportion of T cells (CD4+ and CD8+) as well as B cells in the peripheral blood of mice. Furthermore, the concentrations of inflammatory cytokines, including IL-6, TNF-α, and IFN-γ, substantially decrease following the administration of QFPDD, as reported by Tian *et al.* in 2022^84^.

Treatment with GQD or FMT-GQD helps to replenish the gut microbiota, leading to increased levels of *Akkermansia muciniphila*, *Desulfovibrio C21_c20*, and *Lactobacillus salivarius* while reducing *Escherichia coli* populations. GQD may impact the overall immune response by interacting with the gut microbiota, ultimately providing protection against influenza virus-induced pneumonia in mice^82^. DYY decoction can reduce lung injury caused by RSV through a mechanism that may involve relieving inflammation and regulating gastrointestinal hormone levels, the percentage of lymphocytes and the abundance of beneficial and harmful bacteria in the intestinal tract^105^.

### 3.5 Synergistic multi-target mechanism of TCMs

TCMs involve multiple cell signalling pathways in the treatment of respiratory viruses. Potential targets for novel treatment approaches can be found in these pathways. Antiviral drug targets for TCMs may involve (1) cell signalling pathways that trigger an exaggerated immune response and the release of inflammatory cytokines and (2) various pathways that are suppressed by viruses. Examples include NF-κB signalling, PI3K/Akt signalling, MAPK signalling, and PKC/PKR signalling.

#### 3.5.1 Regulating the NF-κB pathway

The NF-κB pathway plays a crucial role in regulating the production of inflammatory molecules. Following viral infection, the activation of TLR3-mediated MyD88-independent signalling and TLR7-mediated MyD88-dependent signalling leads to the activation of the nuclear transcription factor NF-κB, resulting in the upregulation of proinflammatory factor expression^106^. NF-κB exists in a compound form with its suppressor IκB. For this complex to dissociate, IKK activation is required^107^. When activated, IKK phosphorylates IκB protein, leading to its degradation and resulting in the release of the transcriptionally active NF-kB subunits p65/p50 from the inhibitory complex^108^.

In wild-type mice, Yinqiao powder significantly decreases the expression levels of TLR7, MYD88, IRAK4, and NF-κB, which are elevated during viral infection. This powder affects the TLR7/NF-κB signalling pathway in the context of anti-IFV infection, as reported by Fu *et al.* in 2018^109^. QFPDD inhibits the phosphorylation and activation of the TAK1/IKKα/β/IκB/NF-κB signalling pathway, demonstrating its anti-inflammatory and immunoregulatory effects in response to single-stranded RNA viral infection^71^. JHQG decreases the protein levels of TLR4, MyD88, and p-p65 and the nuclear p65 ratio, indicating that JHQG therapy suppresses the TLR4/MyD88/NF-κB signalling pathway^69^. DYY blocks hypoxia-induced activation of the NF-κB signalling pathway^103^. Liang-Ge-San (LGS) greatly reduces the synthesis of IL-6 and TNF-α in LPS-stimulated RAW 264.7 macrophages; the phosphorylation and degradation of IκBα, as well as the movement of NF-κB p65 into the nucleus, are also prevented. Additionally, LGS triggers the α7 nicotinic acetylcholine receptor (α7nAchR). The inhibition of α7nAchR with the selective inhibitor MLA or α7nAchR siRNA reduces the inhibitory impact of LGS on IκBα, NF-κB p65, IL-6, and TNF-α. LGS has been shown to effectively suppress LPS-induced inflammation in rats with ALI by activating the NF-κB signalling pathway^110^. QFPDD and XFBD have been shown to reduce the expression of inflammatory cytokines, suppress NF-κB signalling pathway activation, and attenuate pinocytosis in macrophages derived from THP-1 cells^111^.

#### 3.5.2 Regulating the PI3K/Akt pathway

Certain PI3K inhibitors or those targeting the downstream signal Akt have the ability to effectively hinder both virus entry and replication, as demonstrated by Pleschka and colleagues in 2001^112^. Patchouli alcohol was shown to effectively block the p-PI3K and p-Akt proteins in cells infected with IAV, with no effect on uninfected A549 cells and no direct enhancement of the *ex vivo* interferon system. This indicates that the inhibitory effect of PA on the PI3K/Akt pathway may be linked to its ability to inhibit IAV infection rather than its direct effects on the host antiviral response^48^. Rhein was found to have a notable inhibitory effect on IAV-induced Akt phosphorylation, although it did not affect the phosphorylation of Akt^59^.

#### 3.5.3 Regulating the MAPK pathway

MAPK pathway activation is involved in the natural antiviral response of cells, but overactivation of this pathway can lead to harmful inflammation in the host^59^. The mRNA levels of Mapk3 (Erk1) and Mapk10 (Jnk3) decrease upon FM1 infection but increase with XDY treatment. A reduction in the mRNA levels of Mapk3 and Mapk10 following FM1 infection could represent a host cell response to the inhibition of the ERK and JNK pathways. XDY can maintain the equilibrium between antiviral defences and inflammatory damage by modulating the activation of ERK and JNK in the MAPK pathway during IAV infection, as excessive activation of these pathways can lead to tissue damage^113^. Rhein has been shown to greatly reduce the phosphorylation of MAPK induced by IAV, although it does not affect the phosphorylation of ERK/MAPK^59^. Pseudoephedrine and its derivative have been found to effectively reduce the phosphorylation of inflammation-related proteins, including NFκB p65, p44/42 MAPK, SAPK/JNK, p38, and IκBα^114^.

#### 3.5.4 Regulating the PKC/PKR pathway

Following viral infection, HA quickly triggers protein kinase C (PKC) and a particular PKC inhibitor. Treatment with XDY has been shown to significantly decrease the expression of phosphorylated p38, ROCK1, phosphorylated MYPT, and phosphorylated PKC caused by IFV infection in pulmonary microvascular endothelial cells. Xuan *et al.* demonstrated that XDY suppresses the influenza-induced reorganization of F-actin in PMVECs by reducing the expression of p-ERM through the inhibition of the Rho/ROCK, p38 MAPK, and PKC pathways^115^.

## 4. Discussion

The constant changes in viruses and the intricate nature of disease-causing processes have long been the main challenges in the study of life sciences. TCMs have demonstrated specific qualities and benefits in preventing and treating viral pneumonia and have become prevalent in various regions due to the frequent occurrence of viral respiratory infections. TCMs have multiple targets and multiple pathways involved in respiratory viral infection intervention, host immune function regulation, organ protection, and host metabolic function regulation. Compared with DNA viruses, RNA viruses such as coronaviruses are more susceptible to errors and mutations during replication. Their high degree of variability makes it more difficult to develop vaccines, and they are more likely to develop resistance to single chemical drugs. Herbal and compound drugs are characterized by a complex network of multiple components, multiple pathways and multiple pathways.

Furthermore, the occurrence of drug resistance is uncommon in the application of TCM in clinical settings. In the diagnostic and therapeutic process, evidence-based treatment is the most effective way to realize the holistic concept of TCM. The ancient text “Huangdi Neijing” established the principle of “different treatments for the same disease” many centuries ago, becoming a fundamental rule in TCM that greatly influenced future medical practices. The term “same treatment for different diseases” is a term proposed by later generations according to the spirit of “different treatment for the same disease” and the actual clinical situation^162^. “Disease” is a general term for all diseases and consists of basic elements such as cause, location, symptom and nature of disease. The term “different treatment for the same disease” refers to the fact that the same disease may be treated differently due to different components of the disease. “Same treatment for different diseases” means that different diseases may be treated in the same way due to the same disease components. MXSGs have the ability to address RSV, IAV, and SARS-CoV-2 infection simultaneously. Additionally, JHQG, DYY and QFPDD can be used to treat SARS-CoV-2 infection. These are the best manifestation of evidence-based treatment with TCMs.

The greatest strength of TCMs is the synergistic effect of multiple components, pathways, and targets. Due to their numerous elements, TCM effects are not dependent on a singular antiviral mechanism but instead involve the simultaneous presence and interplay of various mechanisms. However, insufficient research on the chemical composition and mechanism of action of TCMs hinders the advancement of TCMs and is a clear drawback in contemporary medical systems. There is still a lack of adequate safety studies on the use of TCMs for treating viral illnesses. Hence, for the global advancement of TCMs, assessments of their potential adverse reactions are crucial. In addition, we should search for active substances in TCMs that have better intervention effects on different pathological stages of viral pneumonia, optimize compounding (or structures), and elucidate the mechanism of action. These data could uncover fresh perspectives that have been challenging to uncover through conventional biology, elucidate the mechanisms of herbal remedies against viruses using contemporary life science technology and terminology, and facilitate the seamless fusion of TCM and modern biotechnology.

A comparison between TCM and Western medicine highlights the complementary strengths of both approaches in treating viral pneumonia. While Western medicine provides rapid development of targeted antiviral agents, TCM offers broad-spectrum antiviral activity, especially in the absence of specific antiviral drugs during early outbreaks. As the development of specific antiviral medications is often slow, especially when confronted with emerging and novel viral strains, TCM can be an effective alternative treatment that provides immediate therapeutic options. This is particularly valuable when no effective Western medicine options are available^163^. Additionally, TCM can help reduce infection rates and nucleic acid positivity in high-risk populations, such as the elderly and those with underlying conditions^164^, ^165^.

Furthermore, in treating viral pneumonia, TCM and Western medicine are not only complementary but also can be integrated effectively to enhance patient outcomes. TCM is essential in relieving symptoms like fever, cough, and fatigue in patients with mild viral pneumonia, thereby improving comfort and expediting recovery^27^. TCM treatments have been shown to shorten the time to nucleic acid conversion, effectively reducing the likelihood of progression to severe disease^166^. By managing these early symptoms and improving overall clinical outcomes, TCM can help prevent the disease from worsening^167^.

For severe and critically ill patients, TCM complements conventional Western treatments by improving inflammatory markers and blood oxygen saturation, which can help shorten hospitalization time and reduce mortality rates^164^. Studies have shown that adding TCM to the therapeutic regimen for severe viral pneumonia significantly reduces the rate of progression to severe disease and lowers mortality^168^. Moreover, different TCM formulations, such as herbal injections, are more suitable for severe and critically ill patients due to their rapid absorption and high bioavailability, enhancing their efficacy in these cases^169^, ^170^. Combining TCM with Western medicine has been shown to improve clinical outcomes, reduce severity, and enhance recovery rates^171^.

In the recovery phase, TCM has proven benefits in alleviating residual symptoms and accelerating recovery. TCM is effective in alleviating residual symptoms, accelerating organ function recovery, and improving quality of life. TCM has shown potential in reducing fatigue, breathlessness, and pulmonary fibrosis, as well as promoting immune function and resolving complications such as cytokine storms and myocardial damage^172^. CAM, including TCM, has also demonstrated benefits in managing long COVID symptoms and improving recovery outcomes^173^.

Recent TCM studies have primarily examined IFV, SARS-CoV-2, and RSV in relation to viral pneumonia, with less emphasis on AdV, SARS-CoV, and MERS-CoV. Many CMRIs are not equipped with P3 laboratories (biosafety level 3 laboratories) or higher biosafety laboratories. This greatly limits the extensive and in-depth research on TCM against viral diseases. Fortunately, in 2020, China's Ministry of Science and Technology (MOST) issued the “Guidance on Strengthening the Biosafety Management of Laboratories for High-Level Viral Microorganisms of Novel Coronaviruses”, which requires laboratories to play the role of a platform to serve the needs of scientific and technological research. This will provide strong policy support for in-depth research on the antiviral effects of TCM and its mechanisms. In the future, we need to conduct more research to reveal the combined mechanism of antiviral effects and pharmacological basis of TCMs in treating viral pneumonia. Despite the application of molecular biology techniques in examining the antiviral properties of TCMs, the precise therapeutic impacts of TCMs or compound mixtures on viruses and pneumonia remain unexplored because of their intricate makeup. Hence, research should focus on the combined action mechanism and pharmacodynamic foundation of formulas for treating viral pneumonia utilizing TCM theories and solid clinical evidence. In addition, researchers should also seek to discover active substances in TCMs with potential therapeutic effects on viral pneumonia and their synergistic mechanisms of action. We will search for active substances (including compound components, active parts or monomers) with potential therapeutic effects on different pathological processes of viral pneumonia in TCM and carry out research on their effectiveness, optimized compounding (or structures) and mechanisms of action. TCMs can be used to effectively manage patients with viral pneumonia via evidence-based treatment in a scientific and standardized manner. Due to the anti-viral infection mechanisms of TCM would be conserved in species, no matter of the viral type. Thus, it is the significant advantages for TCM used in new emerging viruses.

TCM of viral pneumonia is based on the overall concept of multiple components synergistically play a role in pharmacological effects, viral suppression, immune balance, organ protection, metabolic regulation and other aspects of the role of advantages, the future can be based on the basis of pharmacological effects of the material basis of the research and development of innovative drugs.

## Supplementary Material

Supplementary table.

## Figures and Tables

**Figure 2:**
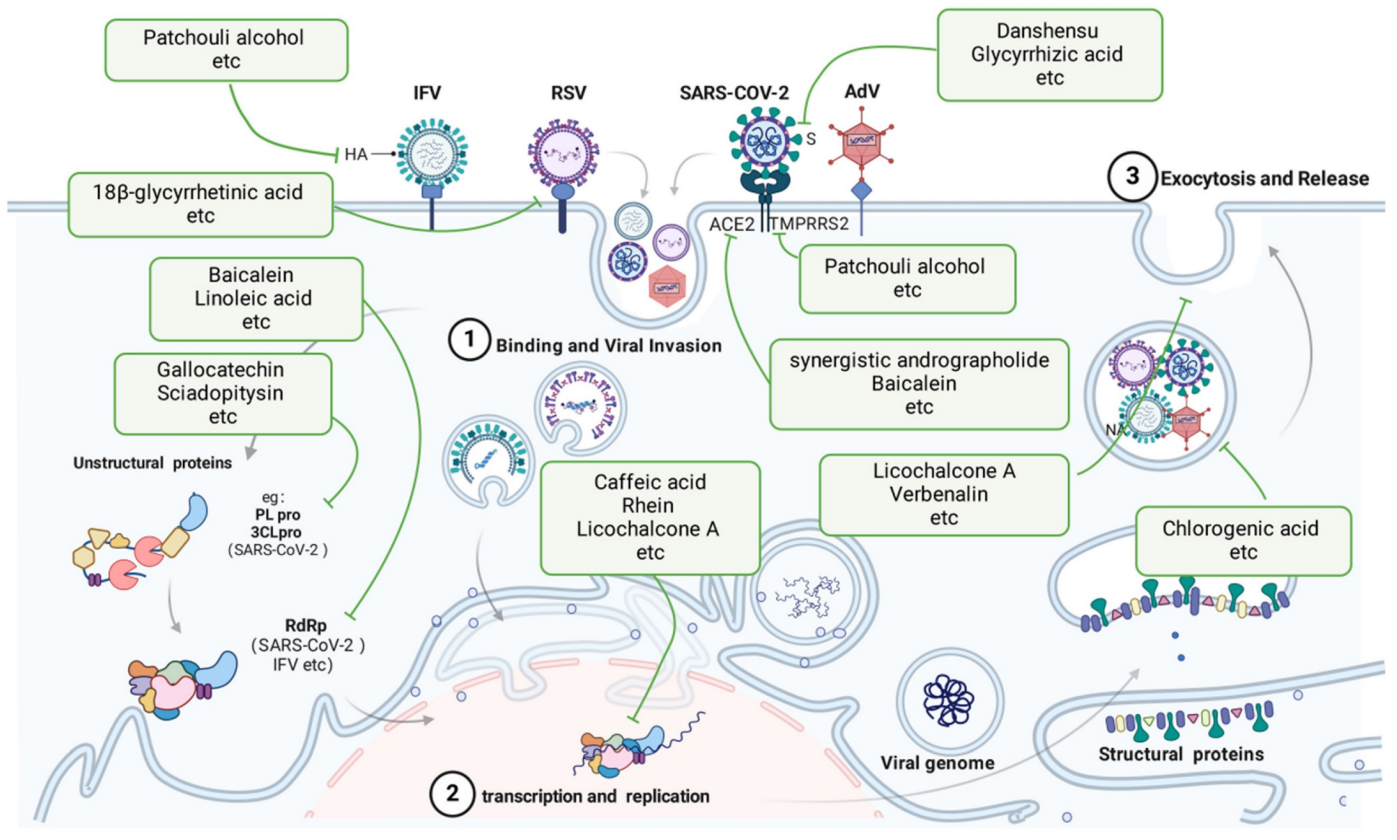
Viral and antiviral action of TCMs. TCMs inhibit viral pneumonia through multiple mechanisms, including binding viruses, blocking viral invasion, transcription and replication, and preventing exocytosis and release. The green boxes list the representative TCMs associated with each mechanism. The illustration was produced using BioRender, a website specializing in scientific graphics.

**Figure 3:**
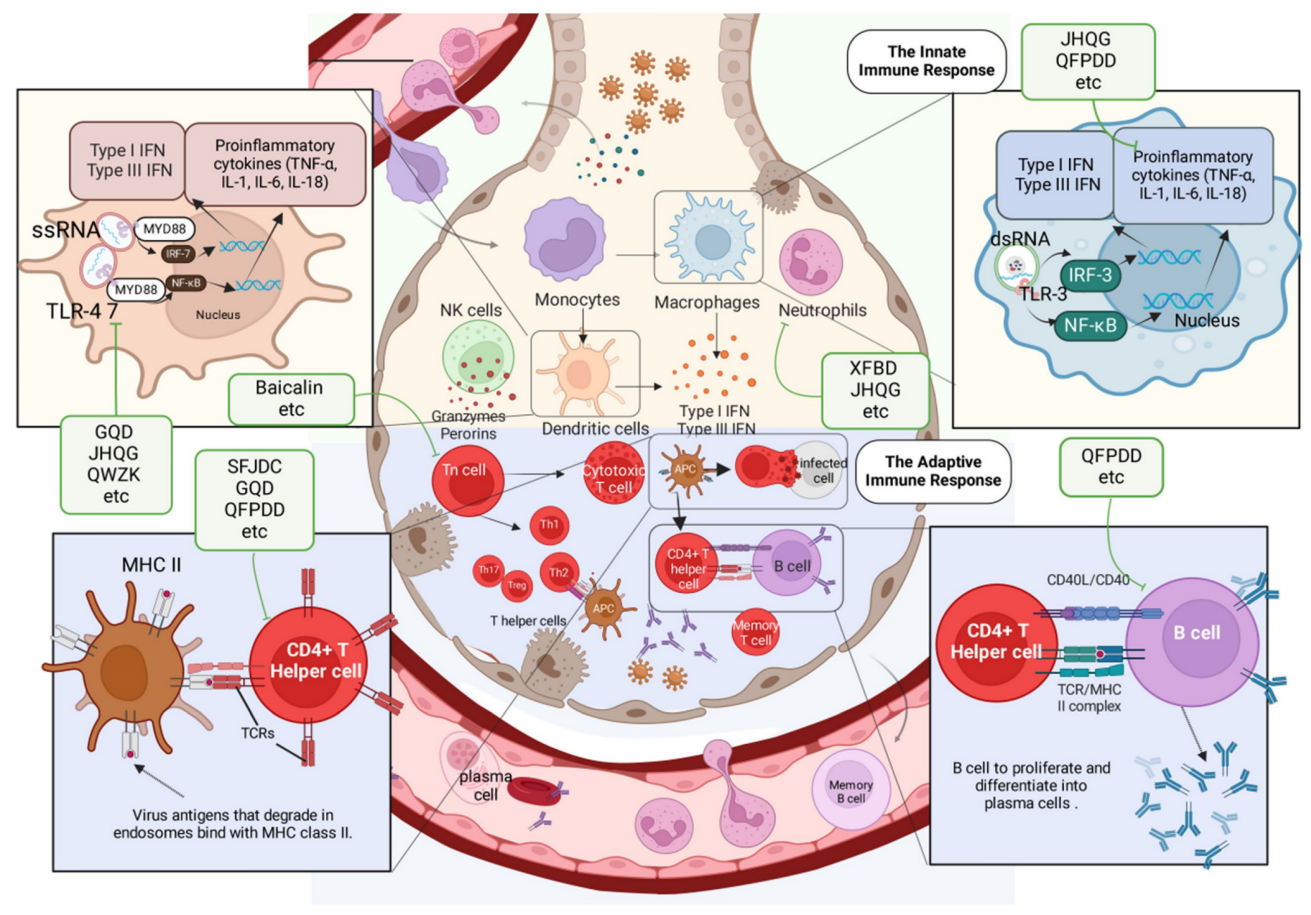
Lung immune cells activated by TCMs. The activation of lung immune cells during viral infection includes both innate and adaptive immunity. Neutrophils and DCs, as part of the innate immune system, trigger adaptive immune responses by secreting IFN, producing antibodies, and killing infected cells. The green boxes list the representative TCMs associated with each mechanism. The illustration was produced using BioRender, a website specializing in scientific graphics.

**Figure 4:**
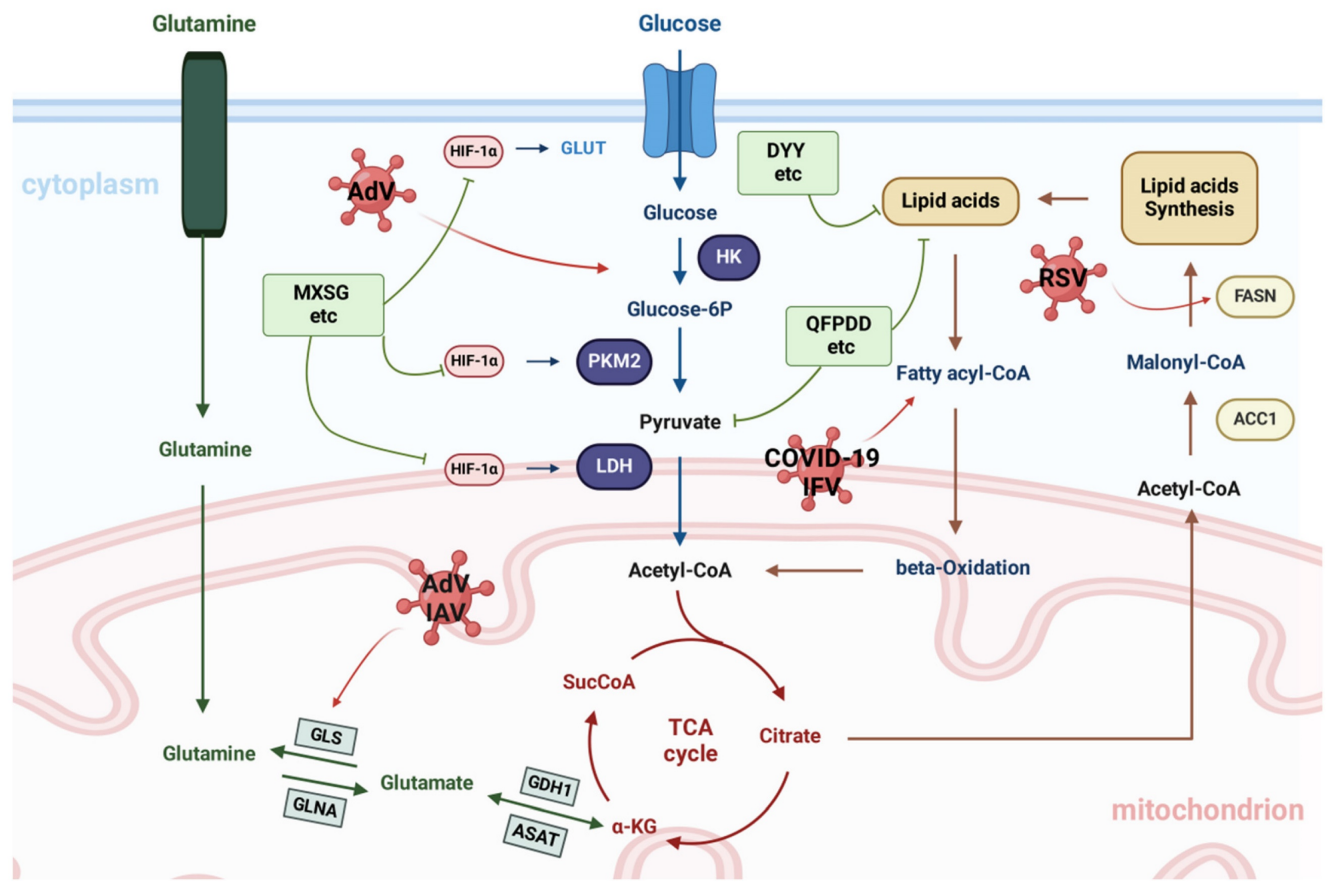
Protection of organs by TCMs. Viruses must breach the epithelial or mucosal barrier to gain entry into the body. They spreads through lymphatic vessels or through the bloodstream (freely or within inflamed cells). This in turn leads to organ damage in the host. The associated damage caused by respiratory viruses in each organ is listed in the red box. The green boxes list the representative TCMs associated with each mechanism. The illustration was produced using BioRender, a website specializing in scientific graphics.

**Figure 5:**
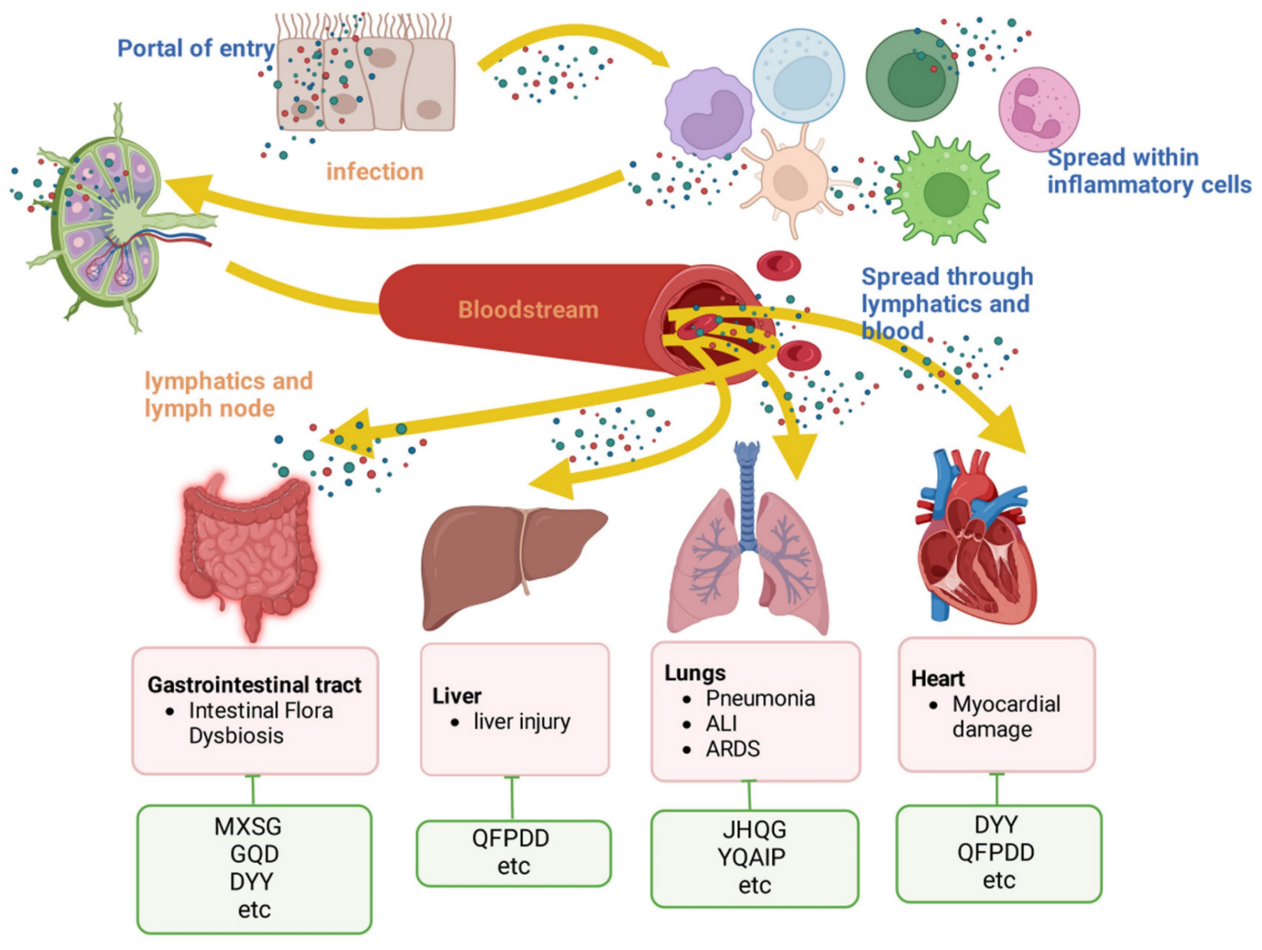
Regulation of metabolism by TCMs. Metabolic pathways are influenced by viral infections and particularly affect the metabolism of glucose, glutamine, and fatty acids in the host. The green boxes list the representative TCMs associated with each mechanism. The illustration was produced using BioRender, a website specializing in scientific graphics.

**Figure 6:**
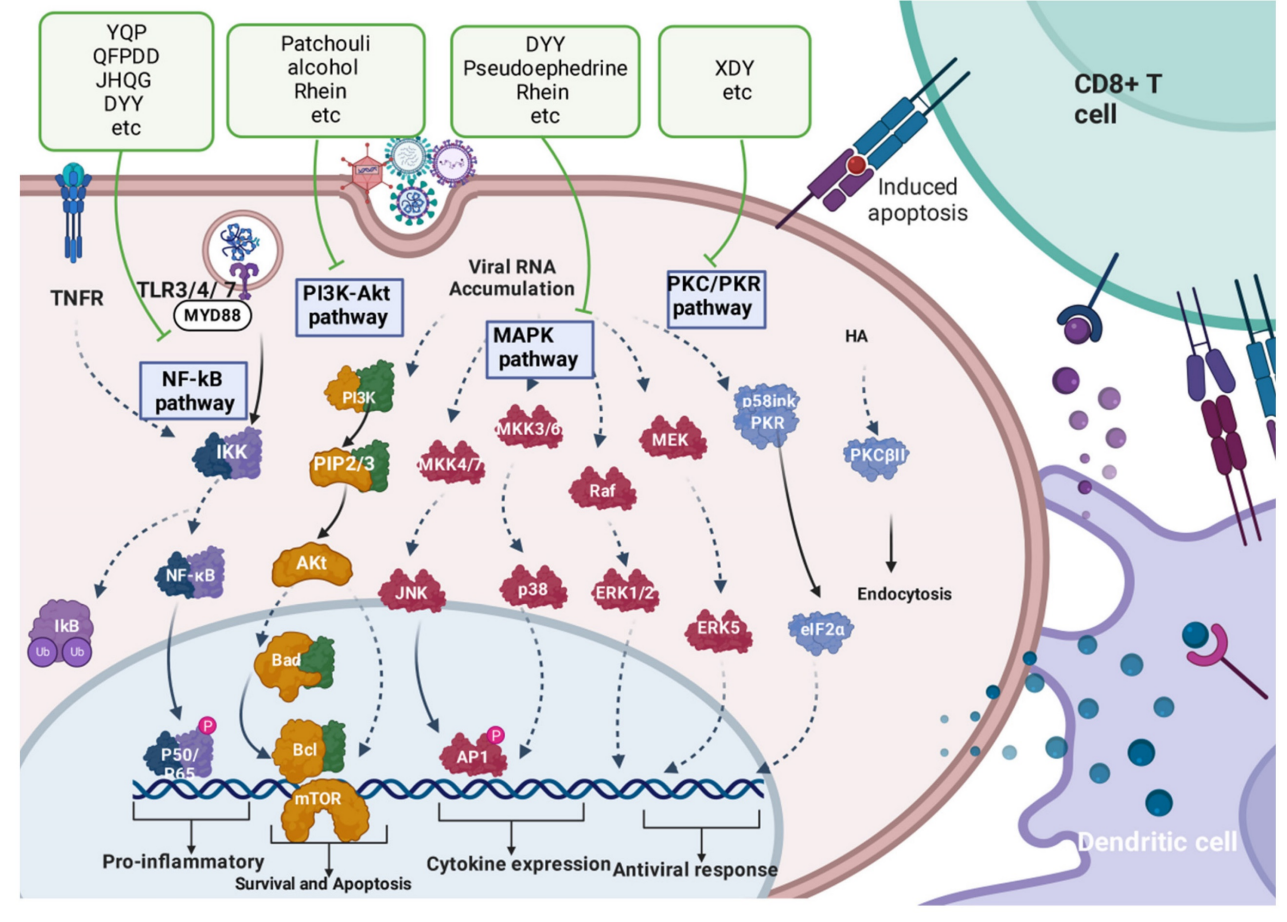
Synergistic multitarget mechanism of TCMs. Cellular pathways in the host that become active during viral lung infection. The green boxes list the representative TCMs associated with each mechanism. The illustration was produced using BioRender, a website specializing in scientific graphics.

**Table 1:** Summary of the classical Chinese herbal formulas used for the treatment of viral pneumonia and their TCM syndrome characteristics.

Virus	Chinese herbal formula	Efficacy-Chinese herbal formula components	
IFV	JHQGQFPDDDYYSFJDCYQAIPGQDYQSMXSGXBCQHSBDLGSLHQW	Wind: Lonicera japonica Thunb., Forsythia suspensa, Fructus Arctii, Menthahaplocalyx, Ephedra sinica Stapf, CassiaTwig, Asarum heterotropoides F. Schmidt, Atractylodes Lancea (Thunb.) DC., Ligusticum chuanxiong hort., Saposhnikovia divaricata (Turcz.) Schischk., Nepeta cataria L., Peucedanum praeruptorumDunn, Notopterygium incisum Ting ex H. T. ChangHeat: Lonicera japonica Thunb., Gypsum, Scutellaria baicalensis Georgi, Forsythia suspensa, Fritillaria thunbergii, Anemarrhena asphodeloides, Fructus Arctii, Artemisia carvifolia, Menthahaplocalyx, Alisma plantago-aquatica Linn., Bupleurum chinense DC., Belamcandachinensis, Glycyrrhizae, Reynoutria japonica Houtt, Lsatis tinctoria, Thlaspi arvense Linn, Phragmitis rhizoma, Puerariae Lobatae Radix, Peucedanum praeruptorumDunn, Cimicifuga foetida L., Coptis chinensis Franch., Lophatherum gracile Brongn, Glycine max (L.) Merr., Rheum palmatum L, Paeoniae Radix Rubra, Gardenia jasminoides J. Ellis, Mirabilite, Lophatherum gracile Brongn, Nakai, Houttuynia cordata ThunbDampness: Scutellaria baicalensis Georgi, Alisma plantago-aquatica Linn., Polyporus umbellatus (Pers.) Fr., Atractylodes macrocephala Koidz., Poria cocos (Schw.) Wolf, Pinellia ternata (Thunb.) Breit., Citrus reticulata Blanco, Agastache rugosa (Fisch.et Mey.) O. Ktze., Magnolia officinalis, Amomum tsaoko Crevost & Lem., Reynoutria japonica Houtt, Atractylodes Lancea (Thunb.) DC., Saposhnikovia divaricata (Turcz.) Schischk., Notopterygium incisum Ting ex H. T. Chang, Coptis chinensis Franch., Rheum palmatum L, Gardenia jasminoides J. EllisCold: Ephedra sinica Stapf, CassiaTwig, Atractylodes Lancea (Thunb.) DC., Zingiber officinale Roscoe, Asarum heterotropoides F. Schmidt, Notopterygium incisum Ting ex H. T. ChangToxin: Lonicera japonica Thunb., Scutellaria baicalensis Georgi, Forsythia suspensa, Fritillaria thunbergii, Fructus Arctii, Glycyrrhizae, Belamcandachinensis, Reynoutria japonica Houtt, Lsatis tinctoria, Thlaspi arvense Linn, Verbena officinalis L., Cimicifuga foetida L., Coptis chinensis Franch., Rheum palmatum L., Gardenia jasminoides J. Ellis, Dryopteris crassirhizoma Nakai, Houttuynia cordata ThunbPhlegm: Fritillaria thunbergii, Fructus Arctii, Glycyrrhiza uralensis Fisch., Pinellia ternata (Thunb.) Breit., Zingiber officinale Roscoe, Aster tataricus L. f., Tussilago farfara Linn, Belamcandachinensis, Citrus aurantium L., Citrus reticulata Blanco, Magnolia officinalis, Amomum tsaoko Crevost & Lem., Reynoutria japonica Houtt, Platycodon grandiflorus (Jacq.) A.DC., Peucedanum praeruptorumDunn, Trichosanthes kirilowii MaximDeficiency: Glycyrrhiza uralensis Fisch., Atractylodes macrocephala Koidz., Poria cocos (Schw.) Wolf, Citrus reticulata Blanco, Dioscorea opposita Thunb, Astragalus membranaceus (Fisch.) Bunge, Rhodiola rosea L., Coix lacryma-jobi L., Atractylodes Lancea (Thunb.) DC., Dryness: Anemarrhena asphodeloides, Paeoniae Radix Alba, Ephedra sinica Stapf (honey-coated, roasted), Amygdalus Communis Vas, Aster tataricus L. f., Tussilago farfara Linn, Phragmitis rhizoma, Puerariae Lobatae Radix, Mirabilite	




SARS-CoV-2	JHQGQFPDDDYY XFBDGQDHSBDLHQW	Wind: Lonicera japonica Thunb., Forsythia suspensa, Fructus Arctii, Menthahaplocalyx, Menthahaplocalyx, Ephedra sinica Stapf, CassiaTwig, Asarum heterotropoides F. Schmidt, Atractylodes Lancea (Thunb.) DC.Heat: Lonicera japonica Thunb., Gypsum, Scutellaria baicalensis Georgi, Forsythia suspensa, Fritillaria thunbergii, Anemarrhena asphodeloides, Fructus Arctii, Artemisia carvifolia, Menthahaplocalyx, Alisma plantago-aquatica Linn., Bupleurum chinense DC., Belamcandachinensis, Paeoniae Radix Alba, Reynoutria japonica Houtt, Phragmitis rhizoma, Puerariae Lobatae Radix, Coptis chinensis Franch., Paeoniae Radix Rubra, Rheum palmatum L., Lsatis tinctoria, Dryopteris crassirhizoma Nakai, Houttuynia cordata Thunb, GlycyrrhizaeDampness: Scutellaria baicalensis Georgi, Alisma plantago-aquatica Linn., Polyporus umbellatus (Pers.) Fr., Atractylodes macrocephala Koidz., Poria cocos (Schw.) Wolf, Pinellia ternata (Thunb.) Breit., Citrus reticulata Blanco, Agastache rugosa (Fisch. et Mey.) O. Ktze., Magnolia officinalis, Amomum tsaoko Crevost & Lem., Coix lacryma-jobi L., Atractylodes Lancea (Thunb.) DC., Reynoutria japonica Houtt, Citri Grandis Exocarpium, Coptis chinensis Franch., Rheum palmatum L.Cold: Zingiber officinale Roscoe, Asarum heterotropoides F. Schmidt, Atractylodes Lancea (Thunb.) DC., Ephedra sinica Stapf, CassiaTwigToxin: Lonicera japonica Thunb., Scutellaria baicalensis Georgi, Forsythia suspensa, Fritillaria thunbergii, Fructus Arctii, Belamcandachinensis, Glycyrrhizae, Coix lacryma-jobi L., Reynoutria japonica Houtt, Verbena officinalis L., Coptis chinensis Franch., Rheum palmatum L., Lsatis tinctoria, Dryopteris crassirhizoma Nakai, Houttuynia cordata ThunbPhlegm: Fritillaria thunbergii, Fructus Arctii, Pinellia ternata (Thunb.) Breit., Zingiber officinale Roscoe, Aster tataricus L. f., Tussilago farfara Linn, Belamcandachinensis, Citrus aurantium L., Citrus reticulata Blanco, Magnolia officinalis, Amomum tsaoko Crevost & Lem., Glycyrrhiza uralensis Fisch., Reynoutria japonica Houtt, Citri Grandis ExocarpiumDeficiency: Glycyrrhiza uralensis Fisch., Atractylodes macrocephala Koidz., Poria cocos (Schw.) Wolf, Citrus reticulata Blanco, Coix lacryma-jobi L., Atractylodes Lancea (Thunb.) DC., Dioscorea opposita Thunb, Astragalus membranaceus (Fisch.) Bunge, Rhodiola rosea L.Dryness: Anemarrhena asphodeloides, Paeoniae Radix Alba, Ephedra sinica Stapf (honey-coated), Amygdalus Communis Vas, Aster tataricus L. f., Tussilago farfara Linn, Phragmitis rhizoma, Puerariae Lobatae Radix	







RSV	DYYSFJDCYQSMXSGLHQW	Wind: Forsythia suspensa, Lonicera japonica Thunb., Menthahaplocalyx, Nepeta cataria L., Ephedra sinica Stapf, Fructus Arctii, MenthahaplocalyxHeat: Anemarrhena asphodeloides, Scutellaria baicalensis Georgi, Reynoutria japonica Houtt, Forsythia suspensa, Lsatis tinctoria, Bupleurum chinense DC., Thlaspi arvense Linn, Phragmitis rhizoma, Glycyrrhizae, Lonicera japonica Thunb., Menthahaplocalyx, Lophatherum gracile Brongn, Glycine max (L.) Merr., Fructus Arctii, Gypsum, Dryopteris crassirhizoma Nakai, Houttuynia cordata Thunb, Rheum palmatum L.Dampness: Magnolia officinalis, Amomum tsaoko Crevost & Lem., Scutellaria baicalensis Georgi, Reynoutria japonica Houtt, Agastache rugosa (Fisch. et Mey.) O. Ktze., Rheum palmatum L.Cold: Ephedra sinica StapfToxin: Scutellaria baicalensis Georgi, Reynoutria japonica Houtt, Forsythia suspensa, Lsatis tinctoria, Thlaspi arvense Linn, Verbena officinalis L., Glycyrrhiza uralensis Fisch., Lonicera japonica Thunb., Fructus Arctii, Dryopteris crassirhizoma Nakai, Houttuynia cordata Thunb, Rheum palmatum L.Phlegm: Magnolia officinalis, Amomum tsaoko Crevost & Lem., Reynoutria japonica Houtt, Glycyrrhiza uralensis Fisch., Platycodon grandiflorus (Jacq.) A.DC., Fructus ArctiiDeficiency: Glycyrrhiza uralensis Fisch., Rhodiola rosea L.Dryness: Anemarrhena asphodeloides, Ephedra sinica Stapf (roasted), Amygdalus Communis Vas	







AdV	SFJDCYQSMXSGLHQW	Wind: Forsythia suspensa, Lonicera japonica Thunb., Menthahaplocalyx, Nepeta cataria L., Ephedra sinica Stapf, Fructus Arctii, MenthahaplocalyxHeat: Reynoutria japonica Houtt, Forsythia suspensa, Lsatis tinctoria, Bupleurum chinense DC., Thlaspi arvense Linn, Phragmitis rhizoma, Lonicera japonica Thunb., Menthahaplocalyx, Lophatherum gracile Brongn, Glycyrrhizae, Glycine max (L.) Merr., Fructus Arctii, Gypsum, Dryopteris crassirhizoma Nakai, Houttuynia cordata Thunb, Rheum palmatum L.damp: Reynoutria japonica Houtt, Agastache rugosa (Fisch. et Mey.) O. Ktze., Rheum palmatum L.Cold: Ephedra sinica StapfToxin: Reynoutria japonica Houtt, Forsythia suspensa, Lsatis tinctoria, Thlaspi arvense Linn, Verbena officinalis L., Lonicera japonica Thunb., Glycyrrhiza uralensis Fisch., Fructus Arctii, Dryopteris crassirhizoma Nakai, Houttuynia cordata Thunb, Rheum palmatum L.Phlegm: Reynoutria japonica Houtt, Platycodon grandiflorus (Jacq.) A.DC., Glycyrrhiza uralensis Fisch., Fructus ArctiiDeficiency: Glycyrrhiza uralensis Fisch., Rhodiola rosea L.Dryness: Ephedra sinica Stapf (roasted), Amygdalus Communis Vas	








Abbreviations: DYY: Dayuanyin, GQD: Gegen Qinlian decoction, LHQW: Lianhuaqingwen capsule, JHQG: Jinhua Qinggan granules, LGS: Liang-Ge-San, MXSG: Maxing shigan decoction, QFPD: Qingfei Paidu decoction, QWZ: Qingwenzhike prescription, SFJDC: Shufeng Jiedu capsules, XFBD: Xuanfei Baidu Decoction, XDY: Xijiao Dihuang decoction combined with Yinqiao powder, YQAIP: Yinqiao Anti-infective Powder, YQP: Yinqiao powder.

**Table 2:** Summary of the historical origin and clinical application of classical Chinese herbal formulas for viral pneumonia

Chinese herbal formula	Earliest recorded dynasty	Book	Efficacy and treatment (TCM)	Clinical trial
DYY	Ming	《On Plague Diseases》	Reconciling shaoyang	SARS-CoV-2☆^8^ IFV☆^9^
GQD	Han	《Treatise on Cold Pathogenic and Miscellaneous Diseases》	Relieving both superficial and internal disorders	SARS-CoV-2☆^10^ IFV☆^11^, ^12^
LHQW	Qing	《Detailed Analysis of Epidemic Warm Diseases》	Relieving superficies syndrome with pungent	SARS-CoV-2△^13^-^15^ IFV☆^13^, ^16^ RSV☆^17^, ^18^
JHQG	Qing	《Detailed Analysis of Epidemic Warm Diseases》	Relieving superficies syndrome with pungent	SARS-CoV-2☆^19^, ^20^ IFV☆^21^ AdV☆^22^
LGS	Song	《Prescriptions of the Bureau of Taiping People's Welfare Pharmacy》	Clearing heat and removing toxicity	SARS-CoV-2☆^23^, ^24^ IFV△^25^
MXSG	Han	《Treatise on Cold Pathogenic and Miscellaneous Diseases》	Relieving superficies syndrome with pungent	SARS-CoV-2☆^26^ IFV☆^27^ RSV☆^28^ AdV△^29^
QFPD	Han	《Treatise on Cold Pathogenic and Miscellaneous Diseases》	Relieving superficies syndrome with pungent	SARS-CoV-2☆^30^-^32^
QWZ	Qing	《Detailed Analysis of Epidemic Warm Diseases》	Relieving superficies syndrome with pungent	SARS-CoV-2☆^33^, ^34^
SFJDC	—	Wisdom from Folk	Clearing heat and removing toxicity	SARS-CoV-2☆^32^, ^35^ IFV☆^36^ RSV△^37^
XFBD	Han	《Treatise on Cold Pathogenic and Miscellaneous Diseases》	Desiccating formula	SARS-CoV-2☆^38^-^40^
XDY	Tang	《Essential Recipes for Emergent Use Worth A Thousand Gold》	Formula for clearing nutrient level and cooling blood	IFV☆^41^
YQAIP	Qing	《Detailed Analysis of Epidemic Warm Diseases》	Relieving superficies syndrome with pungent	IFV,RSV☆^42^, ^43^
YQP	Qing	《Detailed Analysis of Epidemic Warm Diseases》	Relieving superficies syndrome with pungent	SARS-CoV-2☆^44^, ^45^ IFV☆^46^ RSV,AdV△^47^

☆Randomized Controlled Trial, △Case Reports. Abbreviations: DYY: Dayuanyin, GQD: Gegen Qinlian decoction, LHQW: Lianhuaqingwen capsule, JHQG: Jinhua Qinggan granules, LGS: Liang-Ge-San, MXSG: Maxing shigan decoction, QFPD: Qingfei Paidu decoction, QWZ: Qingwenzhike prescription, SFJDC: Shufeng Jiedu capsules, XFBD: Xuanfei Baidu Decoction, XDY: Xijiao Dihuang decoction combined with Yinqiao powder, YQAIP: Yinqiao Anti-infective Powder, YQP: Yinqiao powder.

**Table 3:** Summary of the antiviral pneumonia effects of Chinese herbal formulas and their possible mechanisms of action

Herbal formula	Ingredients	Possible active ingredients	Disease/virus type	Model	Molecular mechanisms and outcomes	Reference
DYY	Arecae SemenPaeoniae Radix AlbaRadix ScutellariaeMagnoliae OfficinalisAnemarrhenae RhizomaAmomum tsao-koRadix Glycyrrhizae Praeparata	QuercetinKaempferolβ-SitosterolBaicaleinAnhydroicaritinStigmasterol	SARS-CoV-2	Hypoxic pulmonary hypertension (HPH) in C57/BL6J mice	②↓MON, percentage of MON in all white blood cells; ↓IL-1β, IL-6, STAT3, NF-κB, PCNA, ERK1/2③↓RVSP, lung index, wall thickness of pulmonary arteries	103
			RSV	Male C57BL/6 mice infected with RSV (ii.)	②↓IL-1β, IL-6 ③↓lung index, regulating the abundance of beneficial and harmful bacteria in the intestinal tract.	105
			RSV	Male C57BL/6 mice infected with RSV (ii.)	②↓IL-1β, IL-6 ③↓lung index, ④regulatory purine metabolism, arachidonic acid metabolism, glycine, serine and threonine metabolism	96
GQD	Radix PuerariaeRadix ScutellariaeRhizoma CoptidisRadix Glycyrrhizae		IAV	C57BL/6 mice infected with IFV FM1	②↓TLR7, MyD88, NF-κB p65, Th1/Th2, Th17/Treg, CD4+	68
			IAV	C57BL/6 mice infected with IFV FM1	②↑claudin-1, ZO-1, occludin, Tregs, ↓NOD1, NOD2, RIP2, NF-κB, IL-10, IL-6, IL-17A, TGF-β, Th17/Treg, CD4+③↓inflammatory cell, lung index	82
			ALI	SD rats administered LPS (ii.)	②↓IL-6, TNF-α, IL-1β, MPO, C3, C5, IL17, TGF-β, CY1A1④↑Firmicutes, ↓Bacteroidetes, ↑acetic, propionic, butyric acid	97
LHQW	Forsythia suspensa (Thunb.) VahlLonicera japonica Thunb. Ephedra sinica StapfArmeniaca sibirica (L.) Lam. Gypsum Fibrosum Dryopteris crassirhizoma Nakai Isatis tinctoria L. Houttuynia cordata Thunb. Pogostemon cablin(Blanco)Benth. Rhodiola rosea L. Rheum palmatum L. Mentha canadaensis L. Glycyrrhiza uralensis Fisch.		IAV (H1N1)	A549 cells infected with PR8BALB/c mice infected with MRSA after PR8 IFV	②↓IL-6, IL-8, TNF-α, CEACAM-1, ICAM-1, VCAM-1, PAFr③↓adhesion of bacteria	116
			SARS-CoV-2	SARS-CoV-2 virus was propagated in Vero E6 cells	①↓plaque, IC 50=411.2 μg/mL②↓TNF-α, IL-6, CCL-2/MCP-1, CXCL-10/IP-10	117
			IBV	MDCK cells infected with B/Guangzhou/GIRD08/2009, B/Guangzhou/GIRD01/2016, B/Guangzhou/0215/2012 and B/Guangzhou/19/2016 female BALB/c mice infected with B/Guangzhou/0215/2012 (ii.)	①IC 50=228±150 to 754±161μg/mL, ↓NP, HA②↓IL-6, IL-8, IP-10, TNF-α, MCP-1 ③↓lung Index	118
JHQG	Glycyrrhiza glabra L.Scutellaria baicalensis Georgi. Forsythia suspensaVahlEphedra sinica StapfLonicera japonica Thunb. Artemisia annua L. Anemarrhena asphodeloides Bunge Prunus sibirica L. Mentha haplocalyx Briq. Arctium lappa L. Fritillaria thunbergii Miq	Luteolin	SARS-CoV-2	Male C57BL/6 mice administered LPS (ii.) RAW 264.7 cells	②↓p-ERK, p-STAT3, TLR4, p-κbα, i-κbα, IL-1β, IL-6, TNF-α ↓macrophages (marker F4/80)③↓lung coefficient, lung wet/dry ratio	72
			IAV (H1N1)	BALB/c mice administered LPS (ip.)	②↓TNF-α, IL-1β, IL-6, Mcl-1, Bcl-xL, caspase-3/7, TLR4, MyD88, p-p65③↓wet-to-dry ratio of the lungs, MPO activity in the lungs and total protein concentration	69
LGS	Forsythia suspenseVahl, Rheum palmatum L.Scutellaria baicalensis GeorgiGardenia jasminoides Ellis. Glycyrrhiza uralensis Fisch. Mentha haplocalyx Briq. Natrii Sulfas.		ALI	LPS-stimulated RAW 264.7 macrophages Male Wistar rats administered LPS (ii.)	②↓IL-6, TNF-α, MPO, MIP-1α, MIP-2, NF-κB p65, p-IκBα③↓lung weight/dry weight ratio, total protein concentration, total cells count, neutrophils count④↑α7nAchR	110
			ALI	Zebrafish administered LPS-yolkLPS-stimulated RAW 264.7 macrophages zebrafish treated with CuSO4 or tail fin injury	②↓IL-6, TNF-α, p-Nur77, p-JNK	119
			ALI	Zebrafish administered LPS-yolkLPS-stimulated RAW 264.7 macrophages	②↓GSK-3β, iNOS ↑MR	120
			ALI	LPS-stimulated RAW 264.7 macrophages	②↓IL-6, TNF-α, IL-1β, P-STAT3, STAT3 ↑miR-21	121
	Ephedra sinicaSemen armeniacae amarumGypsum FibrosumGlycyrrhiza uralensis		IAV	male BALB/C infected with IFV FM1	②↓apoptotic cells, caspase-3, TNF-α, ANGPTL4③↓wet-to-dry ratio of the lungs	122
		ephedrineglycyrrhizic acid	IAV	MLE-12 cells infected with IFV PR8Male BALb/c mice infected with IFV PR8	②↑GPX4, ↓ACSL4, ④↓HIF-1α, iNOS, VEGF	91
			IAV	Male BALb/c mice infected with IFV PR8	②↓IFN-γ, IL-8, JAK1, JAK2, STAT1, IRF9③↓lung index	123
		amygdalinEuchrenoneglycyrrhizinglycyrol	SARS-CoV-2	IL-6 induced rat lung epithelial type Ⅱ cells	②↓p-JAK2, p-STAT3, Bax, Caspase 3 ↑Bcl-2	124
			RSV	Male C57BL/6 mice infected with RSV (in.)	②↓IL-4, IL-13, PGE2, SP, TRPV1	101
QFPDD	EphedraRadix glycyrrhizaeAlmondsraw gypsumRamulusCinnamomiRhizoma alismatisPolyporus umbellatusAtractylodes japonica KoidzumiPoriaBupleurumScutellariaGinger-processed PinelliaGingerRadixet Rhizoma AsterisFlos FarfaraeRhizoma BelamcandaeRadix et Rhizoma AsariRhizoma DioscoreaeFructus Aurantii ImmaturusPericarpium Citri ReticulataeHerba Agastachis		ALI	RAW264.7 cellsC57BL/6J mice administered LPS (ii.)	②↑lymphocytes, ↓granulocytes, IL6, IFN-γ, MCP-1, TNF-α, IL-1β, Bax, caspase-3, cleaved caspase-9, C3a, C5a, C5b-9, C5aR, F4/80 ↑Bcl-2③↓wet/dry weight ratio	70
			IAV	C57BL/6J female mice infected with IFV PR8 (in.)	②↓MCP-1, TNF-α, IL-6, IL-1β, MIP-2, MCP-1, IP-10, TAK1, IKK, NF-k B, p65 ↑IL-10③↓ lung index	71
			SARS-CoV-2	BALB/c mice infected with HcoV-229E (in.)	②↑B cell, CD8, CD4 T cells④↓ Staphylococcus, Lachnospiraceae_NK4A136_group, Enterorhabdus, unclassified_f_Lachnospiraceae ↑Alistipes, Odoribacter,	95
			SARS-CoV-2	BALB/c mice infected with HcoV-229E(in.)	②↑CD4, CD8 T cells, B cells ↓IL-6, TNF-α, and IFN-γ ④↑malonic acid, adenosine	84
		Glycyrrhizic acid	ALI	SD rats administered LPS (ii.) LPS-stimulated RAW 264.7 macrophages	②↓IL-6, Poly(I:C)/Pam3CKS4	125
			IAV (myocardial damage)	C57BL/6J female mice infected with IFV PR8(in.)	②↓IFN-β, TNF-α, IL-18 ↓RIPK1, p-RIPK1, RIPK3, p-RIPK3, MLKL, p-MLKL, HIF-1α	104
			SARS-CoV-2	LPS-stimulated A549 and THP-1 cells	②↓IFN-β, TNF-α, IL-6, p-IκBα, p-NF-κB p65	111
		wogonoside	SARS-CoV-2	Dextran sulfate sodium-induced intestinal inflammation in miceintestinal-specific KLHL5 deficient mice	②↓IL-6, TNF-α, ATF2 ↑IL-10	126
QWZK	Ephedrae Herba Gypsum FibrosumRhei Radix Et RhizomaBelamcandae RhizomaAsteris Radix Et RhizomaFarfarae FlosCitri Reticulatae PericarpiumPinelliae Rhizoma Praeparatum Cum Zingibere Et AluminPoriaArmeniacae Semen AmarumCicadae PeriostracumFritillariae Thunbergii BulbusTaraxaci HerbaPlatycodonis Radix	phenolic acid compound-chrysophanol Coumarin compound, emodinTriterpenoids, procyanidin B2,Platycodin D Flavonoids, chlorogenic acidRutinLuteolinOctylgallate	ALI	Wistar mice administered LPS (ii.)	②↓IL-6, TNF-α, MCP-1, IL-1β, IL-18, IFN-γ, TLR4, p-IKKα/β, p-IκBα, p-NF-κB, NLRP3, cleaved caspase-1, ASC③↓WBC count, alveolar wall	33
SFJDC	Polygonum cuspidatumForsythia suspensaIsatis indigoticaBupleurum chinensePatrinia scabiosifoliaVerbena officinalisPhragmites communisGlycyrrhiza uralensis	PolydatinQuercetinWogonin	SARS-CoV-2	BALB/c mice infected with HcoV-229E(in.) SARS-CoV-2 patients	②↓IL-6, IL-10, TNF-α, IFN-γ↑CD4+, CD8+ T Cell, B-Cell③↓lung index, symptomatic (cough and fatigue) period	86
		verbenalinforsythoside Aphillyrinvitexinemodin	ALI	WT C57BL/6 mice infected with PAK LPS-stimulated mouse peritoneal macrophages	②↓IL-6, IL-8, TNF-α	127
			IAV	ICR mice infected with 35 μl of FM1 or PR8 (15LD50) (in.)	③↓lung index, lung index inhibition rate, death rate	128
			IAV	ICR mice infected with 35 μl of FM1 or PR8 (15LD50) (in.)	②↓IFN-γ,TNF-α③↓lung index, lung index inhibition rate, death rate④↑SOD	129
			Viral pneumonia	ICR mice infected with virus (FM1, PR8, B10, B59, HSV-1, HSV-2, RSV, parainfluenza, COX-B4, COX-B5, AV) (in.)ICR mice infected with 35 μl of ParaIFV (15LD50) (in.)	③↓lung index, lung index inhibition rate, death rate	130
			IFV	ICR mice infected with 35 μl of ParaIFV (100TCID50)	②↓IL-6, TNF-α, TLR4, My D88③↓lung index, lung index inhibition rate, death rate	131
XFBD	Ephedra sinica StapfPrunus armeniaca LGypsum fibrosumCoix lacryma-jobi L Atractylodes lanceaPogostemon cablin Artemisia annua LReynoutria japonica HouttVerbena officinalis LPhragmites communis Trin Lepidium apetalum WilldCitrus × reticulata BlancoGlycyrrhiza uralensis Fisch.ex DC.		Pulmonary Fibrosis	TGF-β1 induced fibroblast activation modelLPS/IL-4 induced macrophage inflammation modelMale C57BL/6 mice administered BLM (ii.)	②↓IL-6, STAT3, F4/80, CD206+③↓fibroblast collagen deposition, α-SMA, migration of fibroblasts	73
			ALI	Male BALB/c mice administered CY (ip.)LPS-stimulated RAW 264.7 macrophages and THP-1 monocytes	②↑IgG, IgM, IL-2, IL-4, IL-6, splenic lymphocytes, CD4+, CD8+③↑spleen index, thymus index	132
			ALI	Male C57BL/6 mice administered LPS (ii.)	②↓IL-6, TNF-α, Ly-6G, neutrophils, MPO, H3Cit, PNA, CXCL2	79
		glycyrrhizic acid	ALI	Male C57BL/6 mice administered LPS (ii.)RAW 264.7 macrophages administered LPS	②↓IL-6, TNF-α, IL1-β, iNOS, F4/80, IL17A, Timp1, Muc5ac, Ccl2, Cxcl10③↓wet/dry weight ratio	133
			ALI	Male C57BL/6 mice administered IgG-IC (ii.)	②↓C3a, C5a, C3aR, C5aR, IL-6, IL-1β, TNF-α, MCP-1, p-JAK2, SOCSO, p-STAT3, p-IKKα/β, p-NF-κB p65③↓injured lungs	74
		Sitosterol	Pulmonary Fibrosis	Male C57BL/6J mice administered BLMMLE-12	②↓a-Sma, Collagen I, Vimentin, Cd206, Arg, Ym1, p-SRC	134
		polydatinisoliquiritinacteoside	ALI	C57BL/6 mice administered LPSRAW 264.7 cellsZebrafish tail amputation	②↓IL-6, TNF-α, IL-1β, macrophage activation and migration	135
XDY	dried rehmannia rootbuffalo hornChinese herbaceous peonymoutan barkhoneysuckleForsythiae FructusBalloon Flower rootmintFermented SoybeanHerba lophatheriGreat Burdock Acheneschizonepeta spikeliquorice root		IAV	Male BALB/C mice infected with IFV FM1 (in.)	②↓Fos mRNA (4dpi, 7 dpi), Stat1, Ifnb1 mRNA (4 dpi), ↑Mapk10 mRNA (4dpi, 7 dpi), Mapk3 mRNA (7 dpi)	113
			IAV	Male Wistar rats infected with IFV FM1 (in.)	②↓p-ERM, p-p38, p-MKK, ROCK1, p-MYPT, p-PKC③↑F-actin, ↓T stress fibre formation	115
			IAV	Male BALb/c mice infected with IFV PR8 (in.)J774A.1 cells	②↓IL-1β, nucleoprotein mRNA, NLRP3, CASPASE-1, GSDMD- n, ASC, LC3I, LC3II, P62	41
YQAIP	Paeoniae Radix AlbaMenthae HerbaAtractylodes Lancea (Thunb.) Dc.Radix BupleuriChuanxiong RhizomaSaposhnikoviae Radix Poria Cocos (Schw.) Wolf.Radix PuerariaePogostemon Cablin (Blanco) Benth.Lonicerae Japonicae FlosSchizonepetae HerbaPlatycodon GrandiforusForsythiae FructusEphedra HerbaNotopterygii Rhizoma Et RadixCimicifugae RhizomaPeucedani RadixAnemarrhenae RhizomaGypsum Fibrosum	eugeniinProtohypericin3",8"-Binaringenin	IAV	BALB/c mice infected with A/FM/1/47 (FM1, H1N1) 15LD50 (in.)	②IL-1β, IFN-γ, TNF-α, Lactb mRNA, Pnpt1 mRNA, Mthfd2 mRNA ③ ↓lung index	102
YQP	Flos Lonicerae JaponicaeFructus ForsythiaeRadix PlatycodonisRadix Et Rhizoma GlycyrrhizaeSpicaSchizonepetaeSemen Sojae PraeparatumFructus ArctiiLophatherum GracileMint and Rhizoma Phragmitis		IAV	Male BALB/C mice infected with IFV FM1 (in.)	②↓TLR4, TLR3③↓lung index, viral load	136
			IAV	Wild type and TLR7 KO C57BL/6 mice infected with IFV FM1(in.)	②↓Th17/Treg, TLR7, MyD88, IRAK4, NF-κB③↓lung index, viral load	109

Abbreviations: ip.: intraperitoneal injection; ii.: intratracheal injection; in.: intranasal injection, DYY: Dayuanyin, GQD: Gegen Qinlian decoction, LHQW: Lianhuaqingwen capsule, JHQG: Jinhua Qinggan granules, LGS: Liang-Ge-San, MXSG: Maxing shigan decoction, QFPD: Qingfei Paidu decoction, QWZ: Qingwenzhike prescription, SFJDC: Shufeng Jiedu capsules, XFBD: Xuanfei Baidu Decoction, XDY: Xijiao Dihuang decoction combined with Yinqiao powder, YQAIP: Yinqiao Anti-infective Powder, YQP: Yinqiao powder, Mø: macrophages, BMDMs: bone marrow-derived macrophages, GSDMD: gasdermin D, GSDME: gasdermin E, IL: interleukin, TNF: tumor necrosis factor, PI: propidium iodide, LDH:lactate dehydrogenase, HEK: human embryonic kidney, MASMCs: mouse aorta smooth muscle cells, NHBE: normal human bronchial epithelial, AGMKE: african green monkey kidney epithelial, IC: inhibitory concentration, EC: effective concentration, CC: cytotoxic concentration, MERS: middle east respiratory syndrome. ① ②, ③, and ④ respectively refer to different mechanisms of TCM treatment. ① indicates the interference with respiratory viral infections. ② indicates the regulation of host immune function. ③ indicates the protective effect of organs. ④ indicates regulation of metabolism.

**Table 4:** Summary of the antiviral pneumonia effects of natural compounds and their possible mechanisms of action

Type	Active ingredients	Disease/Virus type	Model	IC50/EC50	Molecular mechanisms and outcomes	Refrence
Alkaloids	Ephedrine	SARS-CoV-2	ACE2 over-expressed HEK293T cellACE2/CMC bioaffinity chromatography model	EC50<20μM	①↓entrance of SARS-CoV-2 spike pseudovirus, ACE2	137
	Pseudoephedrine	SARS-CoV-2	ACE2 over-expressed HEK293T cellACE2/CMC bioaffinity chromatography model	EC50<20μM	①↓entrance of SARS-CoV-2 spike pseudovirus, ACE2	137
		SARS-CoV-2	SARS-CoV-2 S-RBD-LgBiT and SmBiT-ACE2 fusion plasmids transiently co-transfected into HEK293 cells	EC50=13μM	②↓NFκB p65, MAPK, SAPK/JNK, p38, IκBα	114
Anthraquinones	Rhein	ALI	LPS-induced(in.) mouse sepsis model in C57BL/6 mice		②↓IL-6, IL-1β	138
		IAV	C57BL/6J mice infected with H1N1 (in.)MDCK cells and A549 lung cancer cells infected with H1N1	EC50=1.51 μg/mL	①↓IAV adsorption and replication②↓TLR4, Akt, MAPK, NF-κB, p38, JNK, T-SOD, GR, CAT, GSH-PX, MMP③↓Lung Index	59
		RSV	BALB/c mice infected with RSV (in.)		②↓IL-1β, IL-6, TNF-α, IL-18, IL-33, NLRP3, ASC, Caspase-1, p-IκBα, p-NF-κB③↓Lung index	139
Diterpenoids	Andrographolide	SARS-CoV-2	Human lung adenocarcinoma cells infected with SARS-CoV-2		①↓ACE2, S proteins②↓IL-6, TNF-a	55
		RSV	A549 and 16HBE cells infected with RSV		②↑HO-1↓IL-6, IL-8, CXCL10	65
Flavonoids	Baicalin	IAV	C57BL/6 mice infected with H1N1(in.)		↓LDH, caspase-3, Gasdermin ↑Ebubble-like protrusion cells	140
		SARS-CoV-2	Huh7.5 cells infected with SARS-CoV-2 pseudovirus	EC50=9.0μM (Vero cells)EC50=8.0μM (Calu-3 cells)	①↓SARS-CoV-2 replication, SARS-CoV-2 RdRp	61
		IAV	BALB/c mice infected with H1N1(in.)Murine ANA-1 Mø and epithelial BEAS-2B cells infected with H1N1		②↓Recruitment of Mø, M1 polarization, iNOS, TNF-α, ↑IL-1β	141, 142
		IAV	BALB/c mice infected with H1N1(in.)Human lung adenocarcinoma A549 cells infected with H1N1/H3N2	EC50=17.04 µg/ml	②↓miR-146a, TRAF6, IFN-α, IFN-β	143
		IAV	Madin-Darby canine kidney cells infected with H1N1/H3N2/H1N1-H275Y	EC50=4.0μM (H1N1)EC50=2.7μM (H3N2)	①↓IAV replication, neuraminidase	58
		RSV	BALB/c mice infected with RSV (in.)Human epidermoid cancer cells infected with RSV	EC50=19.9μM	①↓RSV G, F,②↓CD4 and CD8 T cells, Mø	64
	Kaempferol	SARS-CoV-2	H-ACE2 mice microinjected with SARS-CoV-2 Luc-VLPs (it.)		①↓invasion of SARS-CoV-2 particles, S2 subunit, HR1 and HR2 of SARS-CoV-2 S2	51
		IFV (H9N2)	MH-S cells infected with H1N1(in.)LPS-induced(ip.) mouse sepsis model in C57BL/6 mice		②↓TNF-α, IL-1β, ROS, TLR-4, NF-κB ③↓Lung Index,	100
	Licochalcone A	RSV	BALB/c mice infected with RSV (in.)		②↓Nrf2, HO-1, IкBα	52
	Licochalcone B	SARS-CoV-2	The African green monkey kidney cells line Vero E6 infected with SARS-CoV-2-related coronaviruses strain GX_P2V	EC50=15.53 µM	①↓viral infection, NP Protein	144
	Curcumin	SARS-CoV-2	Vero E6 infected with SARS-CoV-2PBMCs infected with SARS-CoV-2	EC50=1.14μM (Delta variant)EC50=4.06μM (D614G strain)	②↓IL-6, IL-8, MCP-1, IL-1β	145
		IAV	C57BL/6J mice infected with H1N1(in.)A549 lung cancer cells infected with H1N1		②↑Heme oxygenase-1, IκBα, AMPK, ↓NF-κB	146
		SARS-CoV-2	Mild and severe COVID-19 patients		②↓Th17, IL-17, IL-21, IL-23, G-CSF	147
		IAV	Human Mø or mice infected with H1N1		②↓TNF-α, IFN-α, IL-6, NF-κB, Mø, neutrophils, lymphocytes	148
	Mangiferin	SARS-CoV-2	H292 cells treated with LPSA549 lung cancer cells infected with SARS-CoV-2		②↑IL-10, GSH, ↓IL-6, COX-2, HO-1, TNF-α, MCP-1,③↑wound healing rate	54
Fatty acids	linoleic acid	SARS-CoV-2	C57BL/6 mice expressed human ACE2 receptor.		①↓RdRp of SARS-CoV-2	63
iridoid	Verbenalin	SARS-CoV-2	WT C57BL/6 mice and GPR18-KO mice infected by PAK strain (approximately onetenth of the LD50) (in.)		②↑GPR18-Gi ↓cAMP IL-6, IL-8, TNF-α	127
		SARS-CoV-2	CLP-induced acute lung injury in C57BL/6 mice		②↑EBP-δ, GSDMD, GSDME ↓Mø pyroptosis, NETs	67
Lignans	Honokiol	SARS-CoV-2	Vero E6 and A549 cells infected with SARS-CoV-2	EC50=7.8μM	①↓SARS-CoV-2 replication	62
		SARS-CoV-2	Vero E6 cells infected with SARS-CoV-2	EC50=13μM	①↓SARS-CoV-2 replication	149
Monoterpenoids	Cineole	IAV	BALB/c mice infected with IAV/Font Monmouth/47(H1N1, FM1)		①↓Viral Load②↓IL-4, IL-5, IL-10, MCP-1, IL-6, TNF-α, IL-1β, IFN-γ, ICAM-1, VCAM-1, NF-kB ③↓Lung Index	150
		IAV	BALB/c mice infected with H3N2		②↓IL-10, TNF-α, IL-1β, IFN-γ③↓lung index,viral titers	151
Phenylpropanoids	Ferulate	IAV	BALB/c mice infected with H1N1(in.)MDCK cells infected with H1N1		③↑TLR7/9, MyD88, IRF7↓NF-κB	152
Phenanthraquinones	Tanshinone IIA	IAV	MDCK cells infected with H1N1	EC90=6μg/mL	①↓HA/NA	153
Polyphenols	Resveratrol	SARS-CoV-2	MRC5 cells infected with SARS-CoV-2	EC50=10.66μM		154
		SARS-CoV-2	Vero cells infected with SARS-CoV-2		①↓SARS-CoV-2 mRNA	155
Organic acids	Caffeic acid	IAV	Confluent monolayers of MDCK cells infected with the IFV		①↓multiplication of IAV	57
	Ginkgolic acids	SARS-CoV-2	HEK293, HEK293T cell, NHBE BEAS-2B cells, AGMKE Vero-E6 cells, HEK293F cells, MASMCs infected with SARS-CoV-2 pseudovirus	IC50=28.25-32.54 μMCC50=130.80 μM	①↓SARS-CoV-2 S pseudovirus infection	156
	Ginkgo biloba extract 50	SARS-CoV-2	SARS-CoV-2 3CLpro	IC50=0.98 μM	①↓activity of SARS-CoV-2 3CLpro	60
	Danshensu	SARS-CoV-2	MERS-CoV 3CLpro, HCoV-229E 3CLpro, PLpro and RdRp of SARS-CoV-2	3min: IC50=9.63μM30min: IC50=2.15μM	①↓activity of SARS-CoV-2 3CLpro	157
		SARS-CoV-2	Vero-E6 cells infected with SARS-CoV-2LPS-induced (it.) mouse ALI model in C57BL/6 miceBMDMs	EC50=0.97μMEC50=0.31μM (HEK-293T cells)EC50=4.97μM (Vero-E6 cells)	①↓SARS-CoV-2 S protein-pseudo-typed virus②↓TNF-α, IL-1β, IL-6, CAT, GPx, TLR4, NF-κB p65, AGT ↑ACE2 mRNA③↓thickened alveolar septum, alveoli, inflammatory cell infiltration	50
	Quinoline-2-carboxylic acids	SARS-CoV-2	Human airway epithelial cells (Calu-3) and 293T cells transfected with ACE2	IC50=0.44-1.09 μM	①↓infectivity of SARS-CoV-2 S protein-pseudoviruses, ACE2-RBD interaction	158
	Chlorogenic acid	ALI	LPS-induced(ip.) mouse sepsis model in C57BL/6 mice		②↓IL-6, IL-1β, TNF-α, KAT2A, ac-H3K9	159
		IAV	BALB/c mice infected with H1N1 (in.)MDCK cells infected with H1N1	EC50=44.78μM (H1N1)EC50=62.33μM (H3N2)	①↓NP,②↓IL-6, TNF-α ③↓Lung virus titers, lung index	66
Sesquiterpenes	Patchouli alcohol	IAV	MDCK, Vero cells and A549 lung cancer cells infected with H1N1,H9N2, H3N2,IBVand HSV-1	EC50=19.45μM	①↓HA and NP mRNA, hemolysis of IFV	49
		IAV	MDCK, A549 cells infected with H1N1Kunming mice infected with H1N1(in.)	EC50=3.5-6.3μM	①↓HA and NP mRNA,②↓PI3K, Akt, ERK, MAPK	48
		IAV	16HBE (human respiratory epithelial cells) infected with H1N1		②↓RIG-1, IPS-1, Irf-3, Irf-7, IFN-γ, IL-4, RLH	160
Triterpenoid saponins	Glycyrrhetinic acid	ALI	LPS-induced (in.) mouse sepsis model in C57BL/6 mice		②↓iNOS, TNF-α	161
		RSV	Human epidermoid cancer cells and human lung carcinoma cells infected with RSV	EC50=3.2μM (HEP-2)EC50=5.549μM (A549)	②↑IFN-α, IFN-β	56
Triterpenoid saponins	Glycyrrhizic acid	SARS-CoV-2	Vero E6 cells infected with Spike Protein-Pseudotyped Virus (Lenti-S)		①↓S protein, cell on S protein binding	53

Abbreviations: ip.: intraperitoneal injection; ii.: intratracheal injection; in.: intranasal injection, IL: interleukin, TNF: tumor necrosis factor, TLR: toll-like receptor, RVSP: right ventricular systolic pressure, MON: monocytes, GSDMD: gasdermin D, STAT: signal transducer and activator of transcription, NOD: nucleotide-binding oligomerization domain, RIP: receptor interacting protein, TGF: transforming growth factor, GPx: Glutathione peroxidase, AMP: adenosine monophosphate, IFN: interferon, MCP-1: monocyte chemotactic protein 1, CXCL: C-X-C motif chemokine ligand, p-Nur77: phospho-nuclear hormone receptor77, ANGPTL4: angiopoietin-like 4, IRF: interferon regulatory factor, NLRP: nucleotide-binding oligomerization domain-like receptor family pyrin domain-containing, PCNA: proliferating cell nuclear antigen, CEACAM-1: carcinoembryonic antigen related cell adhesion molecule, ICAM-1: Intercellular cell adhesion molecule-1, VCAM-1: vascular cell adhesion molecule-1, Mcl-1: myeloid cell leukemia-1, Bcl-xL: B-cell lymphoma/lerkemia-xL, TAK1: transforming growth factor-β-activated kinase 1, IKK: inhibitor of kappa B kinase, RIPK1: receptor-interacting protein kinase 1, MLKL: mixed-lineage kinase domain-like, HIF-1α: hypoxia inducible factor-1α, SOD: superoxide dismutase, MPO: myeloperoxidase, PNA: peptide nucleic acid, α-SMA: α-smooth muscle actin, ROCK1: Rho associated coiled coil containing protein kinase 1, MYPT: myosin phosphatase-targeting, PKC: protein kinase C, LC3: microtubule associated protein 1 light chain 3. ① ②, ③, and ④ respectively refer to different mechanisms of TCM treatment. ① indicates the interference with respiratory viral infections. ② indicates the regulation of host immune function. ③ indicates the protective effect of organs. ④ indicates regulation of metabolism.
