# Supplementary material for: Traditional Chinese Medicine for Viral Pneumonia Therapy: Pharmacological Basis and Mechanistic Insights
Source: Int J Biol Sci. 2025 Jan 6;21(3):989–1013. doi: 10.7150/ijbs.105086 (PMC11781171; doi:10.7150/ijbs.105086)
Supplement: Supplementary file 1 — Supplementary table. [file ijbsv21p0989s1.pdf]

Table S1. Summary of potential drugs and Chinese herbal medicines against viruses

Virus type	Target and Mechanism	Drug Candidate	Potential Natural Compounds
IFV	M2 blocker	Amantadine*[1]<br>Rimantadine*[1]	Brevilin A[2]
	NA inhibitor	Oseltamivir*#[1, 3]<br>Zanamivir*#[1, 3]<br>Peramivir*#[1, 3]	Aloin[4]<br>Phenanthrenes(analogues 4)[5]<br>Socorilagin[6]<br>Theaflavin-3,3'-DG[7]<br>Berberine-piperazine derivatives (analogsBPD-13)[8]<br>Pentagalloylglucose[2]<br>Kaempferol[9]<br>Nepein Hispidulin Rosmarinic acid methylester[10]<br>Amurensin K (+)-viniferol C Trans-vitisin B[11]
	RdRP inhibitor	Favipiravir△[12]<br>Ribavirin[13]<br>L-742,001△[14]<br>S-033188△[14]<br>VX-787△[14]	/
	HA inhibitor	Nitazoxanide△[15]<br>DAS181△[16]	Oleanane-type triterpenesaponins 7[17]<br>Theaflavin-3,3'-DG[7]<br>Pentagalloylglucose[2]
	NP inhibitor	Nucleozin[12]<br>Naproxen[1]	Curcumin[1]
	influenza cap-dependent endonuclease inhibitor	Baloxavir marboxil（Early treatment with baloxavir marboxil in high-risk adolescent and adult outpatients with uncomplicated influenza (CAPSTONE-2): a randomised, placebo-controlled, phase 3 trial）	

**SARS-CoV-2**	3CLpro inhibitor	Kaletra[18]<br>Boceprevir[19]<br>Nirmatrelvir☆◇[20-22]<br>Ritonavir☆◇[20-22]	Quercetin[23]<br>Andrographolide[24]<br>Baicalin[25]<br>Luteolin[26]<br>Hesperidin[27]<br>Lignan[28]<br>Betulinic acid[28]<br>Tanshinone[28]<br>Cryptotanshinone[29]<br>Dihydrotanshinone I[29]<br>Tanshinone IIA[29]<br>Curcumin[28]<br>Shikonin[30]
	PLpro inhibitor	Asunaprevir[31]<br>Simeprevir[32]<br>Vaniprevir/Simeprevir[32]<br>Famotidine[33]<br>Ebselen[34]<br>Disulfiram[35]<br>Acriflavine[36]	Sodium Tanshinone IIA Sulfonate[37]<br>Cryptotanshinone[38]<br>Tanshinone I[39]<br>Tanshinone IIA[40]<br>Dihydrotanshinone I[29]
	ACE2 inhibitor	Hydroxychloroquine△[41]<br>Chloroquine△[41]<br>Arbidol△[18]	Emodin[42]<br>Glycyrrhizic acid[43]
	RdRP inhibitor	Ribavirin△[41]<br>Remdesivir△[41]<br>Favipiravir△[41]<br>Nitazoxanide△[41]<br>Veklury*◇[44]<br>Mindvy○[45]	/
	S protein inhibitor	Arbidol△[41]	Emodin[46]<br>Bis-benzylisoquinoline alkaloids-tetrandrine[47]

			Fangchinoline[47]<br>Cepharanthine[47]
	TMPRSS2 inhibitor	Carmustat mesylate△[48]<br>Bromhexine hydrochloride[49]	/
**RSV**	RNA transcription inhibitor	Ribavirin*[50]	/
	Monoclonal antibody	Palivizumab*[51]<br>Motavizumab[52]	/
	F protein inhibitor	ViroPharma[53]<br>JNJ-53718678[54]	Cleistocaltones A[55]<br>Cleistocaltones B[55]
	N protein inhibitor	AstraZeneca[56]	/
**AdV**	DNA replication inhibitor	Cidofovir△[57]<br>Brincidofovir△[57]<br>Filociclovir△[58]	/
	Disruptor of HAdV gene expression	Digoxin[57]<br>Digitoxigenin[57]<br>Lanatoside C[57]<br>Dexamethasone acetate[59]<br>Flunisolide[59]<br>Cytarabine[59]<br>Niclosamide[60]<br>Oxyclozanide[60]<br>Rafoxanide[60]<br>Gemcitabine[61]	/
	Epigenetic regulator-based inhibitor	Chaetocin[62]<br>Lestaurtinib[63]<br>Vorinostat[64]	/
	Nuclear transport inhibitor	Ivermectin[65]<br>Verdinexor[66]	/
	Protease/egress Inhibitor	Nelfinavir[67]	/

CDK Inhibitor	Flavopiridol[68]<br>Roscovitine[69]	/

*Food and Drug Administration, #Centers for Disease Control and Prevention, △clinical trial, ☆Emergency Use Authorization, ◇European Medicine Administration, 〇National Medical Products Administration

References:

[1] Han J, Perez J, Schafer A, et al. Influenza virus: Small molecule therapeutics and mechanisms of antiviral resistance. Curr Med Chem, 2018, 25: 5115-5127

[2] Zhang X, Xia Y, Yang L, et al. Brevilin a, a sesquiterpene lactone, inhibits the replication of influenza a virus in vitro and in vivo. Viruses, 2019, 11:

[3] CDC. Influenza antiviral medications: Summary for clinicians. 2017, 2017(

[4] Huang CT, Hung CY, Hseih YC, et al. Effect of aloin on viral neuraminidase and hemagglutinin-specific t cell immunity in acute influenza. Phytomedicine, 2019, 64: 152904

[5] Shi T, McAllister DA, O'Brien KL, et al. Global, regional, and national disease burden estimates of acute lower respiratory infections due to respiratory syncytial virus in young children in 2015: A systematic review and modelling study. Lancet, 2017, 390: 946-958

[6] Chen F, Yang L, Huang Y, et al. Isocorilagin, isolated from canarium album (lour.) raeusch, as a potent neuraminidase inhibitor against influenza a virus. Biochem Biophys Res Commun, 2020, 523: 183-189

[7] Zu M, Yang F, Zhou W, et al. In vitro anti-influenza virus and anti-inflammatory activities of theaflavin derivatives. Antiviral Res, 2012, 94: 217-224

[8] Enkhtaivan G, Kim DH, Park GS, et al. Berberine-piperazine conjugates as potent influenza neuraminidase blocker. Int J Biol Macromol, 2018, 119: 1204-1210

[9] Jeong HJ, Ryu YB, Park SJ, et al. Neuraminidase inhibitory activities of flavonols isolated from rhodiola rosea roots and their in vitro anti-influenza viral activities. Bioorg Med Chem, 2009, 17: 6816-6823

[10] Bang S, Quy Ha TK, Lee C, et al. Antiviral activities of compounds from aerial parts of salvia plebeia r. Br. J Ethnopharmacol, 2016, 192: 398-405

[11] Nguyen TNA, Dao TT, Tung BT, et al. Influenza a (h1n1) neuraminidase inhibitors from vitis amurensis. Food Chemistry, 2011, 124: 437-443

[12] Yen HL. Current and novel antiviral strategies for influenza infection. Curr Opin Virol, 2016, 18: 126-134

[13] Cheung PP, Watson SJ, Choy KT, et al. Generation and characterization of influenza a viruses with altered polymerase fidelity. Nat Commun, 2014, 5: 4794

[14] Koszalka P, Tilmanis D, Hurt AC. Influenza antivirals currently in late-phase clinical trial. Influenza Other Respir Viruses, 2017, 11: 240-246

[15] Haffizulla J, Hartman A, Hoppers M, et al. Effect of nitazoxanide in adults and adolescents with acute uncomplicated influenza: A double-blind, randomised, placebo-controlled, phase 2b/3 trial. Lancet Infect Dis, 2014, 14: 609-618

[16] Zenilman JM, Fuchs EJ, Hendrix CW, et al. Phase 1 clinical trials of das181, an inhaled sialidase, in healthy adults. Antiviral Res, 2015, 123: 114-119

[17] Mair CE, Grienke U, Wilhelm A, et al. Anti-influenza triterpene saponins from the bark of burkea africana. J Nat Prod, 2018, 81: 515-523

[18] Sanders JM, Monogue ML, Jodlowski TZ, et al. Pharmacologic treatments for coronavirus disease 2019 (covid-19): A review. Jama, 2020, 323: 1824-1836

[19] Choy KT, Wong AY, Kaewpreedee P, et al. Remdesivir, lopinavir, emetine, and homoharringtonine inhibit sars-cov-2 replication in vitro. Antiviral Res, 2020, 178: 104786

[20] Administration USFaD. Coronavirus (covid-19) update: Fda authorizes first oral antiviral for treatment of covid-19. 2021, 2021(December 22):

[21] Pfizer. Paxlovid: Epar - product information. 2023, 2023(January 8):

[22] Administration USFaD. Paxlovid loa 02012023. 2024, 2024(March 13):

[23] Wu W, Li R, Li X, et al. Quercetin as an antiviral agent inhibits influenza a virus (iav) entry. Viruses, 2015, 8:

[24] Yu B, Dai CQ, Jiang ZY, et al. Andrographolide as an anti-h1n1 drug and the mechanism related to retinoic acid-inducible gene-i-like receptors signaling pathway. Chin J Integr Med, 2014, 20: 540-545

[25] Su H, Yao S, Zhao W, et al. Discovery of baicalin and baicalein as novel, natural product inhibitors of sars-cov-2 3cl protease in vitro. bioRxiv, 2020,

[26] Ryu YB, Jeong HJ, Kim JH, et al. Biflavonoids from torreya nucifera displaying sars-cov 3cl(pro) inhibition. Bioorg Med Chem, 2010, 18: 7940-7947

[27] Lin CW, Tsai FJ, Tsai CH, et al. Anti-sars coronavirus 3c-like protease effects of isatis indigotica root and plant-derived phenolic compounds. Antiviral Res, 2005, 68: 36-42

[28] Wen CC, Kuo YH, Jan JT, et al. Specific plant terpenoids and lignoids possess potent antiviral activities against severe acute respiratory syndrome coronavirus. J Med Chem, 2007, 50: 4087-4095

[29] Park JY, Kim JH, Kim YM, et al. Tanshinones as selective and slow-binding inhibitors for sars-cov cy steine proteases. Bioorg Med Chem, 2012, 20: 5928-5935
[30] Zhenming Jin , Xiaoyu Du , Yechun Xu , et al. Structure-based drug design, virtual screening and hi gh-throughput screening rapidly identify antiviral leads targeting covid-19. bioRxiv, 2020,
[31] Anson BJ, Chapman ME, Lendy EK, et al. Broad-spectrum inhibition of coronavirus main and papa in-like proteases by hcv drugs. Res Square (Preprint), 2020,
[32] Gammeltoft KA, Zhou Y, Duarte Hernandez CR, et al. Hepatitis c virus protease inhibitors show dif ferential efficacy and interactions with remdesivir for treatment of sars-cov-2 in vitro. Antimicrob Agen ts Chemother, 2021, 65: e0268020
[33] Wu C, Liu Y, Yang Y, et al. Analysis of therapeutic targets for sars-cov-2 and discovery of potential d rugs by computational methods. Acta Pharm Sin B, 2020, 10: 766-788
[34] Weglarz-Tomczak E, Tomczak JM, Talma M, et al. Identification of ebselen and its analogues as po tent covalent inhibitors of papain-like protease from sars-cov-2. Sci Rep, 2021, 11: 3640
[35] Sargsyan K, Lin CC, Chen T, et al. Multi-targeting of functional cysteines in multiple conserved sars -cov-2 domains by clinically safe zn-ejectors. Chem Sci, 2020, 11: 9904-9909
[36] Napolitano V, Dabrowska A, Schorpp K, et al. Acriflavine, a clinically approved drug, inhibits sars-c ov-2 and other betacoronaviruses. Cell Chem Biol, 2022, 29: 774-784.e778
[37] Xu Y, Chen K, Pan J, et al. Repurposing clinically approved drugs for covid-19 treatment targeting s ars-cov-2 papain-like protease. Int J Biol Macromol, 2021, 188: 137-146
[38] Zhao Y, Du X, Duan Y, et al. High-throughput screening identifies established drugs as sars-cov-2 pl pro inhibitors. Protein Cell, 2021, 12: 877-888
[39] Lim CT, Tan KW, Wu M, et al. Identifying sars-cov-2 antiviral compounds by screening for small mo lecule inhibitors of nsp3 papain-like protease. Biochem J, 2021, 478: 2517-2531
[40] Fang ZY, Zhang M, Liu JN, et al. Tanshinone iia: A review of its anticancer effects. Front Pharmacol, 2020, 11: 611087
[41] Zhang Q, Xiang R, Huo S, et al. Molecular mechanism of interaction between sars-cov-2 and host cells and interventional therapy. Signal Transduct Target Ther, 2021, 6: 233
[42] Schwarz S, Wang K, Yu W, et al. Emodin inhibits current through sars-associated coronavirus 3a pr otein. Antiviral Res, 2011, 90: 64-69
[43] Yu S, Zhu Y, Xu J, et al. Glycyrrhizic acid exerts inhibitory activity against the spike protein of sars-c ov-2. Phytomedicine, 2021, 85: 153364
[44] Administration USFaD. Fda approves first treatment for covid-19. 2020,
[45] Biosciences J. Junshi biosciences announces approval for marketing of vv116 in china. 2023, 2023 (January 29):
[46] Ho TY, Wu SL, Chen JC, et al. Emodin blocks the sars coronavirus spike protein and angiotensin-co nverting enzyme 2 interaction. Antiviral Res, 2007, 74: 92-101
[47] Kim DE, Min JS, Jang MS, et al. Natural bis-benzylisoquinoline alkaloids-tetrandrine, fangchinoline, and cepharanthine, inhibit human coronavirus oc43 infection of mrc-5 human lung cells. Biomolecule s, 2019, 9:
[48] Hoffmann M, Kleine-Weber H, Schroeder S, et al. Sars-cov-2 cell entry depends on ace2 and tmpr ss2 and is blocked by a clinically proven protease inhibitor. Cell, 2020, 181: 271-280.e278
[49] Lucas JM, Heinlein C, Kim T, et al. The androgen-regulated protease tmprss2 activates a proteolyti c cascade involving components of the tumor microenvironment and promotes prostate cancer metast asis. Cancer Discov, 2014, 4: 1310-1325
[50] Ventre K, Randolph AG. Ribavirin for respiratory syncytial virus infection of the lower respiratory t ract in infants and young children. Cochrane Database Syst Rev, 2007, Cd000181
[51] Alansari K, Toaimah FH, Almatar DH, et al. Monoclonal antibody treatment of rsv bronchiolitis in y oung infants: A randomized trial. Pediatrics, 2019, 143:
[52] Ramilo O, Lagos R, Sáez-Llorens X, et al. Motavizumab treatment of infants hospitalized with respi ratory syncytial virus infection does not decrease viral load or severity of illness. Pediatr Infect Dis J, 20 14, 33: 703-709
[53] Kim YI, Pareek R, Murphy R, et al. The antiviral effects of rsv fusion inhibitor, mdt-637, on clinical i solates, vs its achievable concentrations in the human respiratory tract and comparison to ribavirin. Inf luenza Other Respir Viruses, 2017, 11: 525-530
[54] Roymans D, Alnajjar SS, Battles MB, et al. Therapeutic efficacy of a respiratory syncytial virus fusio n inhibitor. Nat Commun, 2017, 8: 167
[55] Song JG, Su JC, Song QY, et al. Cleistocaltones a and b, antiviral phloroglucinol-terpenoid adducts f rom cleistocalyx operculatus. Org Lett, 2019, 21: 9579-9583

[56] Chapman J, Abbott E, Alber DG, et al. Rsv604, a novel inhibitor of respiratory syncytial virus replication. Antimicrob Agents Chemother, 2007, 51: 3346-3353
[57] Naesens L, Lenaerts L, Andrei G, et al. Antiadenovirus activities of several classes of nucleoside and nucleotide analogues. Antimicrob Agents Chemother, 2005, 49: 1010-1016
[58] Toth K, Hussein ITM, Tollefson AE, et al. Filociclovir is a potent in vitro and in vivo inhibitor of human adenoviruses. Antimicrob Agents Chemother, 2020, 64:
[59] Saha B, Varette O, Stanford WL, et al. Development of a novel screening platform for the identification of small molecule inhibitors of human adenovirus. Virology, 2019, 538: 24-34
[60] Marrugal-Lorenzo JA, Serna-Gallego A, Berastegui-Cabrera J, et al. Repositioning salicylanilide anthelmintic drugs to treat adenovirus infections. Sci Rep, 2019, 9: 17
[61] Gray SG, Baird AM, O'Kelly F, et al. Gemcitabine reactivates epigenetically silenced genes and functions as a DNA methyltransferase inhibitor. Int J Mol Med, 2012, 30: 1505-1511
[62] Greiner D, Bonaldi T, Eskeland R, et al. Identification of a specific inhibitor of the histone methyltransferase su(var)3-9. Nat Chem Biol, 2005, 1: 143-145
[63] Hexner EO, Serdikoff C, Jan M, et al. Lestaurtinib (cep701) is a jak2 inhibitor that suppresses jak2/stat5 signaling and the proliferation of primary erythroid cells from patients with myeloproliferative disorders. Blood, 2008, 111: 5663-5671
[64] Saha B, Parks RJ. Histone deacetylase inhibitor suberoylanilide hydroxamic acid suppresses human adenovirus gene expression and replication. J Virol, 2019, 93:
[65] Tessier TM, Dodge MJ, Prusinkiewicz MA, et al. Viral appropriation: Laying claim to host nuclear transport machinery. Cells, 2019, 8:
[66] Widman DG, Gornisiewicz S, Shacham S, et al. In vitro toxicity and efficacy of verdinexor, an exportin 1 inhibitor, on opportunistic viruses affecting immunocompromised individuals. PLoS One, 2018, 13: e0200043
[67] Georgi F, Kuttler F, Murer L, et al. A high-content image-based drug screen of clinical compounds against cell transmission of adenovirus. Sci Data, 2020, 7: 265
[68] Asghar U, Witkiewicz AK, Turner NC, et al. The history and future of targeting cyclin-dependent kinases in cancer therapy. Nat Rev Drug Discov, 2015, 14: 130-146
[69] Veselý J, Havlicek L, Strnad M, et al. Inhibition of cyclin-dependent kinases by purine analogues. Eur J Biochem, 1994, 224: 771-786
